# Estrogen-related receptor gamma regulates mitochondrial and synaptic genes and modulates vulnerability to synucleinopathy

**DOI:** 10.1038/s41531-022-00369-w

**Published:** 2022-08-18

**Authors:** S. N. Fox, L. J. McMeekin, C. H. Savage, K. L. Joyce, S. M. Boas, M. S. Simmons, C. B. Farmer, J. Ryan, L. Pereboeva, K. Becker, J. Auwerx, S. Sudarshan, J. Ma, A. Lee, R. C. Roberts, D. K. Crossman, A. Kralli, R. M. Cowell

**Affiliations:** 1 Neuroscience Department, Drug Discovery Division, Southern Research, Birmingham, AL 35205 USA; 2 Department of Cell, Developmental and Integrative Biology, University of Alabama at Birmingham, Birmingham, AL 35294 USA; 3 Department of Psychiatry and Behavioral Neurobiology, University of Alabama at Birmingham, Birmingham, AL 35294 USA; 4 NeuroInitiative, LLC, Jacksonville, FL 32207 USA; 5 Department of Pediatrics, Infectious Disease, Neuroscience Vector and Virus Core, University of Alabama at Birmingham, Birmingham, AL 35223 USA; 6 Department of Neurodegenerative Science, Van Andel Institute, Grand Rapids, MI 49503 USA; 7 Swiss Federal Institute of Technology Lausanne, Lausanne, Switzerland; 8 Department of Urology, University of Alabama at Birmingham, Birmingham, AL 35294 USA; 9 Department of Genetics, University of Alabama at Birmingham, Birmingham, AL 35294 USA; 10 Department of Physiology, Johns Hopkins University School of Medicine, Baltimore, MD 21205 USA

**Keywords:** Parkinson's disease, Cellular neuroscience

## Abstract

Many studies implicate mitochondrial dysfunction as a key contributor to cell loss in Parkinson disease (PD). Previous analyses of dopaminergic (DAergic) neurons from patients with Lewy-body pathology revealed a deficiency in nuclear-encoded genes for mitochondrial respiration, many of which are targets for the transcription factor estrogen-related receptor gamma (*Esrrg*/ERRγ). We demonstrate that deletion of ERRγ from DAergic neurons in adult mice was sufficient to cause a levodopa-responsive PD-like phenotype with reductions in mitochondrial gene expression and number, that partial deficiency of ERRγ hastens synuclein-mediated toxicity, and that ERRγ overexpression reduces inclusion load and delays synuclein-mediated cell loss. While ERRγ deletion did not fully recapitulate the transcriptional alterations observed in postmortem tissue, it caused reductions in genes involved in synaptic and mitochondrial function and autophagy. Altogether, these experiments suggest that ERRγ-deficient mice could provide a model for understanding the regulation of transcription in DAergic neurons and that amplifying ERRγ*-*mediated transcriptional programs should be considered as a strategy to promote DAergic maintenance in PD.

## Introduction

Parkinson’s disease (PD) is a neurodegenerative movement disorder characterized by the loss of dopaminergic (DAergic) neurons in the substantia nigra pars compacta (SNc) and the development of alpha-synuclein-containing aggregates called Lewy-bodies and Lewy-neurites^1^. Clinical manifestations of motor symptoms are not obvious until ~80% of SNc DAergic neurons are already lost^2^. Current therapies focus on symptom management and dopamine (DA) replacement strategies; however, the beneficial effects of medication deteriorate over time, eventually leading to dyskinesia. A deeper understanding of the mechanisms underlying DAergic neuron susceptibility is needed to generate disease-modifying therapies for PD.

DAergic neurons are particularly vulnerable to oxidative stress and mitochondrial dysfunction^3–9^, and recent studies have shown a progressive, levodopa (L-DOPA)-responsive phenotype in mice in which the catalytic core of complex I of the electron transport chain (ETC) was deleted (*Ndufs2*) selectively in DAergic neurons (MCI-Park mice)^10^. Further evidence suggests that during PD progression, transcriptional programs for the expression of genes involved in mitochondrial structure and function are disrupted^11^. Laser-captured microdissected (LCM) DAergic neurons from postmortem tissue of patients with Lewy-pathology revealed a deficiency in expression for nuclear-encoded genes involved in mitochondrial respiration and function^12^. The concerted decrease in expression for a large number of nuclear-encoded mitochondrial genes suggests that the transcriptional activity of upstream regulators is impaired; in fact, these genes are targets of the transcriptional coactivator and master regulator of transcription of mitochondrial genes peroxisome proliferator-activated receptor gamma coactivator-1 alpha (PGC-1α)^12^. PGC-1α dysfunction and/or deficiency has been linked to PD and to many other neurodegenerative disorders^13–23^. Several studies suggest that targeting PGC-1α to improve mitochondrial function could be a potential therapeutic strategy. However, due to the number of transcription factors with which PGC-1α interacts, manipulating this coactivator may cause unwanted secondary effects (reviewed in McMeekin et al.^14^). It is important to identify the transcription factors involved in regulation of these genes to reveal potential avenues for modulating transcriptional programs that could be dysfunctional in PD.

Direct regulators of nuclear-encoded mitochondrial genes are members of the estrogen-related receptor (ERR) family, encoded by *Esrra*, *Esrrb* and *Esrrg*. Members of this family show sequence homology with estrogen receptors but are incapable of being bound by estrogen. All three factors have been shown to interact with PGC-1α to drive genes involved in mitochondrial structure and function^24–29^; however, *Esrrb* is expressed early in development^26^, and nuclear-encoded mitochondrial genes are not significantly reduced in the midbrain brain of mice lacking *Esrra*^29^, leading us to explore potential roles for *Esrrg* in the brain. Of note, over half of the mitochondrial transcripts reduced in DAergic neurons from patients with Lewy-body pathology are direct transcriptional targets of *Esrrg*^12,26^. ERRγ is expressed in the brain and in DAergic neurons^26,30^, but its roles in neurons are relatively unknown^31–35^. Recent studies have shown a positive association between ERRγ expression levels and that of tyrosine hydroxylase (TH) and dopamine transporter (DAT) in DAergic nerve terminals in vivo and in vitro^36,37^. Altogether, these findings suggest a role for ERRγ in DAergic neurons and highlight its potential as a transcription factor for the regulation of mitochondrial gene expression in DAergic neurons.

Here, we explored whether ERRγ is necessary for normal gene expression in DAergic neurons and whether manipulation of its expression could influence vulnerability to synucleinopathy, with the prediction that its deletion could generate transcriptional and behavioral phenotypes similar to those observed in PD. We show that, while deletion of ERRγ selectively in DAergic neurons does not fully mimic transcriptional alterations in postmortem tissue, it impacts neuronal gene expression, survival, and motor function that is rescuable with L-DOPA. Furthermore, cell type-specific manipulation of ERRγ expression influences DAergic neuron vulnerability in the pre-formed fibril model of synucleinopathy. Transcriptional analyses revealed that ERRγ is required for maintaining mitochondrial content and the expression of genes related to autophagy, mitochondrial respiration and metabolism, synaptic vesicle cycling, transcriptional regulation and vesicle-mediated transport. Conversely, ERRγ overexpression can decrease synuclein load, delay cell-loss, maintain DAergic terminal integrity, and increase expression of transcripts involved in mitochondrial respiration, DA metabolism, and autophagy. Overall, these data indicate that *Esrrg* is required for DAergic gene expression and viability and may serve as a potential target for promoting resilience of DAergic neurons to disease.

## Results

### Putative targets of ERRγ are reduced in Parkinson’s disease

**Fig. 1:**
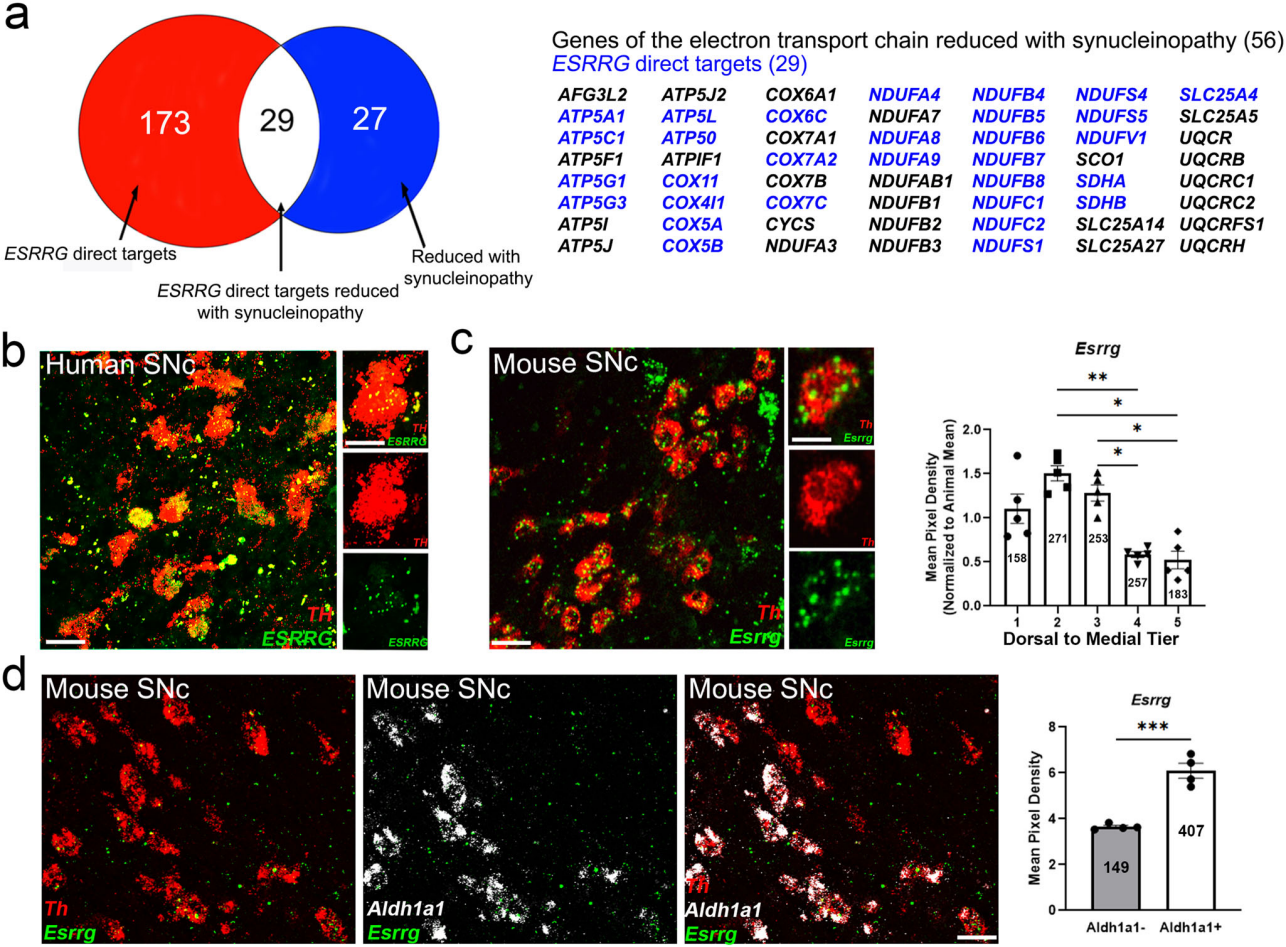
ERRγ targets ETC genes reduced with synucleinopathy and is expressed in DAergic neurons. **a** Of the 56 genes that are reduced with synucleinopathy^12^ and expressed in dopaminergic neurons^120^, 29 genes are also direct targets of *ESRRG*^26^. sm-FISH in human (**b**) or mouse (**c**) SNc for both *Esrrg* (green*)* and *Th* (red) transcripts demonstrate expression of *Esrrg* in DAergic neurons. sm-FISH for *Esrrg* transcript was quantified in *Th*-positive neurons from the dorsal tier to the medial tier of the SNc in mice (*n* = 5 mice/group repeated measures one-way ANOVA with Tukey’s post hoc analysis *p < 0.05, **p < 0.01). **d** sm-FISH for *Esrrg* (green)*, Th* (red), and *Aldh1a1* (white) shows *Esrrg* is more highly expressed in the *Aldh1a*+ population compared to the *Aldh1a1−* population (*n* = 3 mice/group; two-tailed unpaired *t*-test ***p < 0.001). Numbers on bars are total cell counts from each group of an experiment. Scale bars correspond to 50 µm (**b**, **c**) and 10 µm (**b**, **c**). Error bars represent ±SEM.

To explore the potential for ERRγ as an upstream regulator of mitochondrial genes reduced in PD, we cross-referenced ERRγ targets identified by chromatin immunoprecipitation in cultured neurons^26^ with mitochondrial genes reduced in LCM neurons from patients with Lewy-body pathology^12^. We found an overlap of 29 ERRγ targets out of a total of 56 genes, most of which encode for proteins of the ETC (Fig. 1a). *Esrrg* mRNA expression was confirmed in both human (Fig. 1b) and mouse (Fig. 1c) tyrosine-hydroxylase (*Th*)-positive DAergic neurons of the SNc using single molecule fluorescent in situ hybridization (sm-FISH), indicating that *Esrrg* is expressed in the neuroanatomical location to cell-autonomously control mitochondrial gene expression. Interestingly, *Esrrg* was more abundant in DAergic neurons in the dorsal tier of the SNc as compared to the ventral tier (Fig. 1c, as measured by semi-quantitative sm-FISH; mean pixel density = relative area of cell occupied by signal), with highest expression in neurons projecting to the striatum, as labeled by the sub-population marker *Aldh1a1*^38–40^ (Fig. 1d).

### Deletion of *Esrrg* from dopaminergic neurons causes motor deficits, cell loss, and reduction in mitochondrial number

**Fig. 2:**
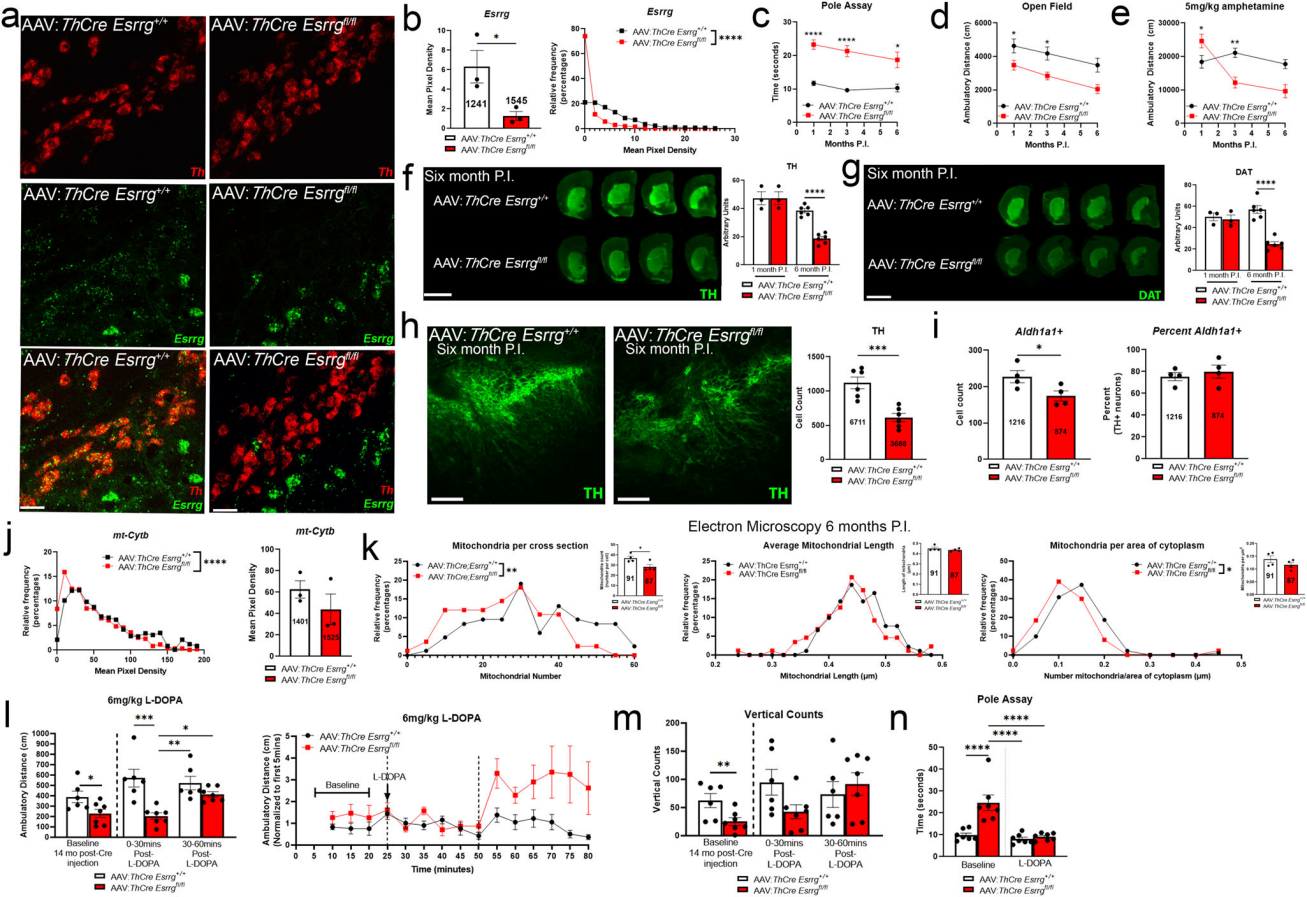
Adulthood deletion of *Esrrg* from DAergic neurons causes motor impairment, cell loss, and a reduction in mitochondrial number. **a** sm-FISH for *Esrrg* (green) and *Th* (red) transcript in *Esrrg*^*+/+*^ and *Esrrg*^*fl/fl*^ mice injected with AAV:*ThCre* into the midbrain, quantified in **b** (*n* = 4 mice/genotype; two-tailed unpaired *t*-test *p < 0.05 or unpaired nonparametric Kolmogorov–Smirnov test ****p < 0.0001). **c**–**e** Ambulatory behavior in *Esrrg*^*+/+*^ and *Esrrg*^*fl/fl*^ mice injected with AAV^:^*ThCre* (1 month *n* = 21 mice/genotype; 3 months *n* = 18–17 mice/genotype; 6 months *n* = 7 mice/genotype; mixed-effects analysis with Sidak’s post hoc analysis *p < 0.05, **p < 0.01, ****p < 0.0001). **f**, **g** TH and DAT immunoreactivity in the striatum of mice 6 months post-injection (P.I.) (*n* = 3/genotype 1-month P.I.; *n* = 6/genotype 6 months P.I., mixed-effects analysis with Sidak’s post hoc analysis ****p < 0.0001). **h** TH immunoreactivity in the SNc 6 months P.I. (*n* = 6 mice/group; two-tailed unpaired *t*-test ***p < 0.001). **i** sm-FISH for *Aldh1a1*+ populations at 6 months P.I. (*n* = 4 mice/group; two-tailed unpaired *t*-test *p < 0.05). **j** sm-FISH for mitochondrially encoded gene *cytb* in mice 1 month P.I. (*n* = 4 per group; two-tailed unpaired *t*-test or unpaired nonparametric Kolmogorov–Smirnov test ****p < 0.0001). **k** Electron microscopy in neurons stained with TH and deficient in *Esrrg* at 6 months P.I. (*n* = 6 mice/group; two-tailed unpaired *t*-test *p < 0.05 or unpaired nonparametric Kolmogorov–Smirnov test *p < 0.05, **p < 0.01). **l**–**n** Ambulatory distance, vertical counts, and pole-assay in *Esrrg*^*+/+*^ and *Esrrg*^*fl/fl*^ mice 14 months P.I. AAV:*ThCre* into the midbrain at baseline and after acute injection of L-DOPA (*n* = 7 mice/group; two-way ANOVA with Tukey’s post hoc analysis *p < 0.05, **p < 0.01, ***p < 0.001). Numbers on bars are cell counts from each experiment. Scale bars correspond to 50 µm (**b**),100 µm (**h**), and 200 µm (**f**, **g**). Error bars represent ±SEM.

**Fig. 3:**
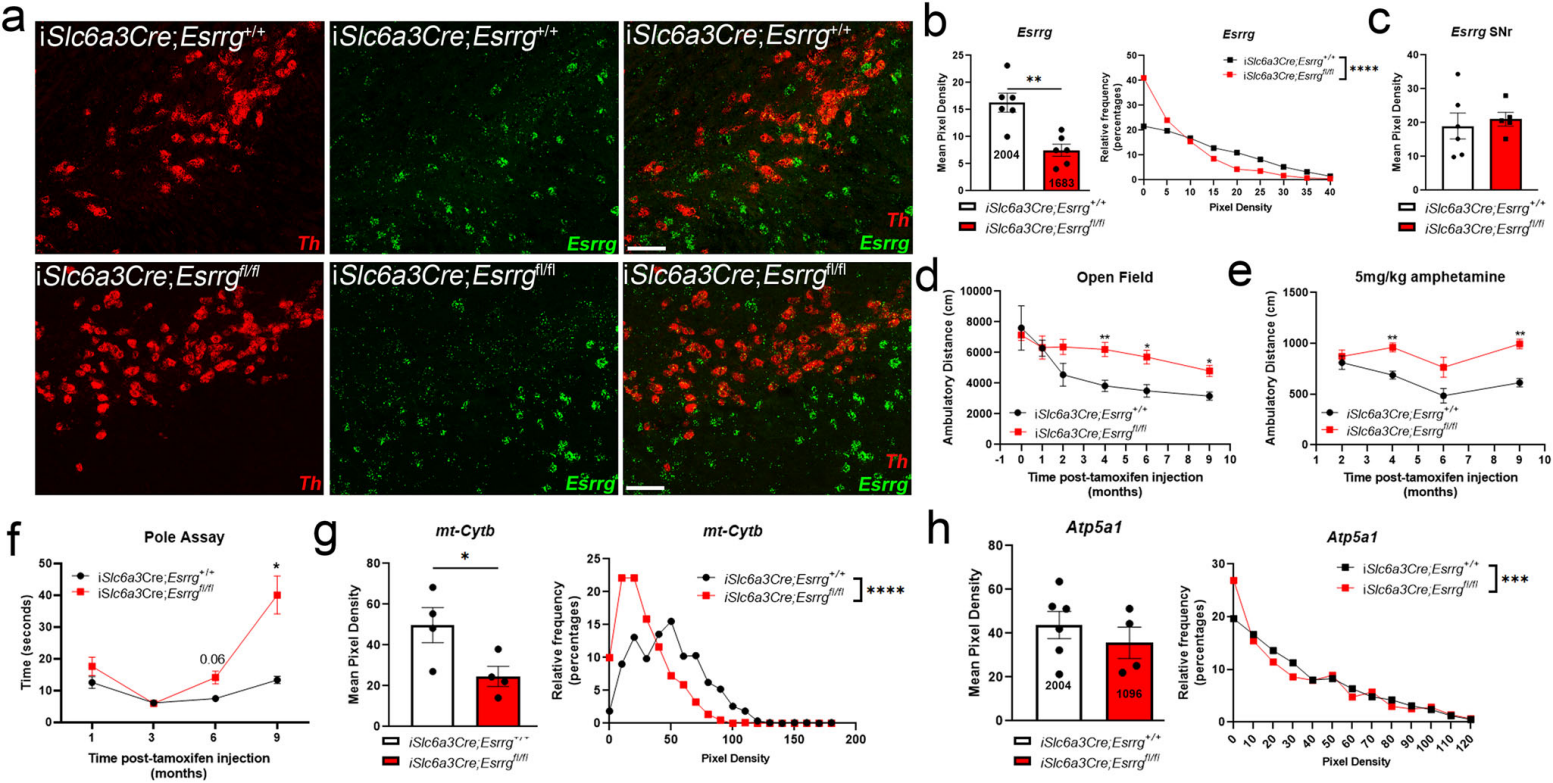
Partial reduction in *Esrrg* expression in adult DAergic neurons using an inducible Cre causes different phenotypes than complete lack of *Esrrg*. **a**, **b** sm-FISH for *Esrrg* (green) and *Th* (red) transcript in i*Slc6a3Cre*;*Esrrg*^*+/+*^ and i*Slc6a3Cre*;*Esrrg*^*fl/fl*^ mice (*n* = 4 mice/genotype; two-tailed unpaired *t*-test *p < 0.05, or unpaired nonparametric Kolmogorov–Smirnov test ****p < 0.0001). **c** Quantification of *Esrrg* by sm-FISH in substantia nigra pars reticulata (SNr). **d**–**f** Ambulatory and pole assay behavior up to 9 months post-tamoxifen injection (12 months of age) (*n* = 5–13 mice/group; mixed-effects analysis with Sidak’s post hoc analysis *p < 0.05, **p < 0.01). **g** sm-FISH for the mitochondrially encoded gene *Cytb* (*n* = 4/group; two-tailed unpaired *t*-test or unpaired nonparametric Kolmogorov–Smirnov test *p < 0.05, ****p < 0.0001). **h** sm-FISH for the nuclear-encoded mitochondrial gene *Atp5a1* (*n* = 4/group; two-tailed unpaired *t*-test or unpaired nonparametric Kolmogorov–Smirnov test ***p < 0.001). Numbers on bars are cell counts from each experiment. Scale bars correspond to 50 µm **a**. Error bars represent ±SEM.

To determine if ERRγ is required for the expression of mitochondrial genes and whether *Esrrg* deletion from DAergic neurons is sufficient to cause a PD-like phenotype in mice, we used two approaches to reduce *Esrrg* in adult mice: (1) nigral injection of adeno-associated virus encoding Cre recombinase driven by the *Th* promoter (AAV-*ThCre*) into mice harboring loxp sites flanking exon 2 of *Esrrg* (DNA-binding domain, deletion causes instability of protein^32,41,42^; Fig. 2) or (2) the cross-breeding of mice expressing an inducible form of Cre recombinase under control of the *Slc6a3* promoter^43^ with *Esrrg*^*fl/fl*^ mice (Fig. 3). Adulthood deletion was important for exploring PD-relevant phenotypes, considering the possible roles for *Esrrg* in developing neurons^26^ and the evidence for early postnatal lethality in whole body *Esrrg* knockout mice^35^. In the first set of experiments, 3 month-old *Esrrg*^*+/+*^ and *Esrrg*^*fl/fl*^ mice were injected bilaterally with AAV9:*ThCre* into the SNc. A > 80% reduction in *Esrrg* mRNA in DAergic neurons of the SNc (Fig. 2a, b) was observed 1 month post-injection, as assessed using sm-FISH probes specific for exon 2 of *Esrrg*. Distribution analyses of single-cell expression values showed a reduction in *Esrrg* expression across the entire SNc DAergic population (Fig. 2b).

To study the functional consequences of this deletion, pole assay and open field tests were used to assess motor coordination and ambulation, respectively (all behavioral results are summarized in Supplementary Table 1, with results split by sex in Supplementary Fig. 1). *Esrrg*^*fl/fl*^ mice injected with AAV:*ThCre* showed a pole assay deficit (Fig. 2c) and a hypoactive phenotype 1, 3, and 6 months post-injection (Fig. 2d), with an early hyperactive response to an injection of *d*-amphetamine (AMPH, Fig. 2e). These behavioral changes were accompanied by a reduction in the staining intensity for TH and DAT in the striatum at 6 months post-injection with no changes in immunoreactivity in the olfactory tubercle or cortex (Fig. 2f, g and Supplementary Fig. 2a, d), and a 50% reduction in SNc DAergic cell bodies quadruple-labeled for TH, NeuN, DAPI, cresyl violet (Fig. 2h). sm-FISH analyses of *Aldh1a1*-positive *Th*-positive neurons at the 6-month post-injection time point revealed that cell death was occurring to a similar extent in *Aldh1a1*-expressing and non-expressing DAergic populations, with a reduction in the total number of *Aldh1a1*-positive neurons but no change in percent of the population (Fig. 2i and Supplementary Fig. 3a).

As an initial readout of modulation of *Esrrg*-dependent gene programs, we confirmed that *Esrrg* deficiency caused a reduction in the expression, per cell, of the mitochondrial-encoded *mt-Cytb* (Fig. 2j and Supplementary Fig. 3b), the transcription of which is driven by the ERRγ-responsive gene *Tfam*^34,44^. Distribution analysis of *mt-Cytb* revealed that the frequency of low *mt-Cytb* expressing cells was increased in the *Esrrg*^*fl/fl*^ mice (Fig. 2j). The reduction in *Esrrg* transcript was accompanied by a reduction in mitochondrial number per DAergic neuron cross-section by electron microscopy with no change in mitochondrial length compared to control (Fig. 2k). There was also a reduction in the number of mitochondria per area of cytoplasm in cell cross-sections from *Esrrg*^*fl/fl*^ compared to *Esrrg*^*+/+*^ mice, with no change in perimeter of either the nucleus or the neuron (Fig. 2k and Supplementary Fig. 3h, i).

To understand if these behavioral phenotypes were responsive to DA-replacement therapy similar to PD patients and other mouse models of PD, aged AAV-*ThCre Esrrg*^*+/+*^ and *Esrrg*^*fl/fl*^ mice were injected with L-DOPA after confirming a hypoactive phenotype in open field (Fig. 2l). An acute dose of L-DOPA (6 mg/kg) was sufficient to rescue ambulation, vertical counts, and pole assay deficits (Fig. 2l, m), without having any effect on wildtype mice, confirming that deletion of *Esrrg* selectively from DAergic neurons is sufficient to cause a L-DOPA-responsive, PD-like phenotype.

### Partial knockdown of *Esrrg* in dopaminergic neurons causes ambulatory hyperactivity and reductions in mitochondrial gene expression

**Fig. 4:**
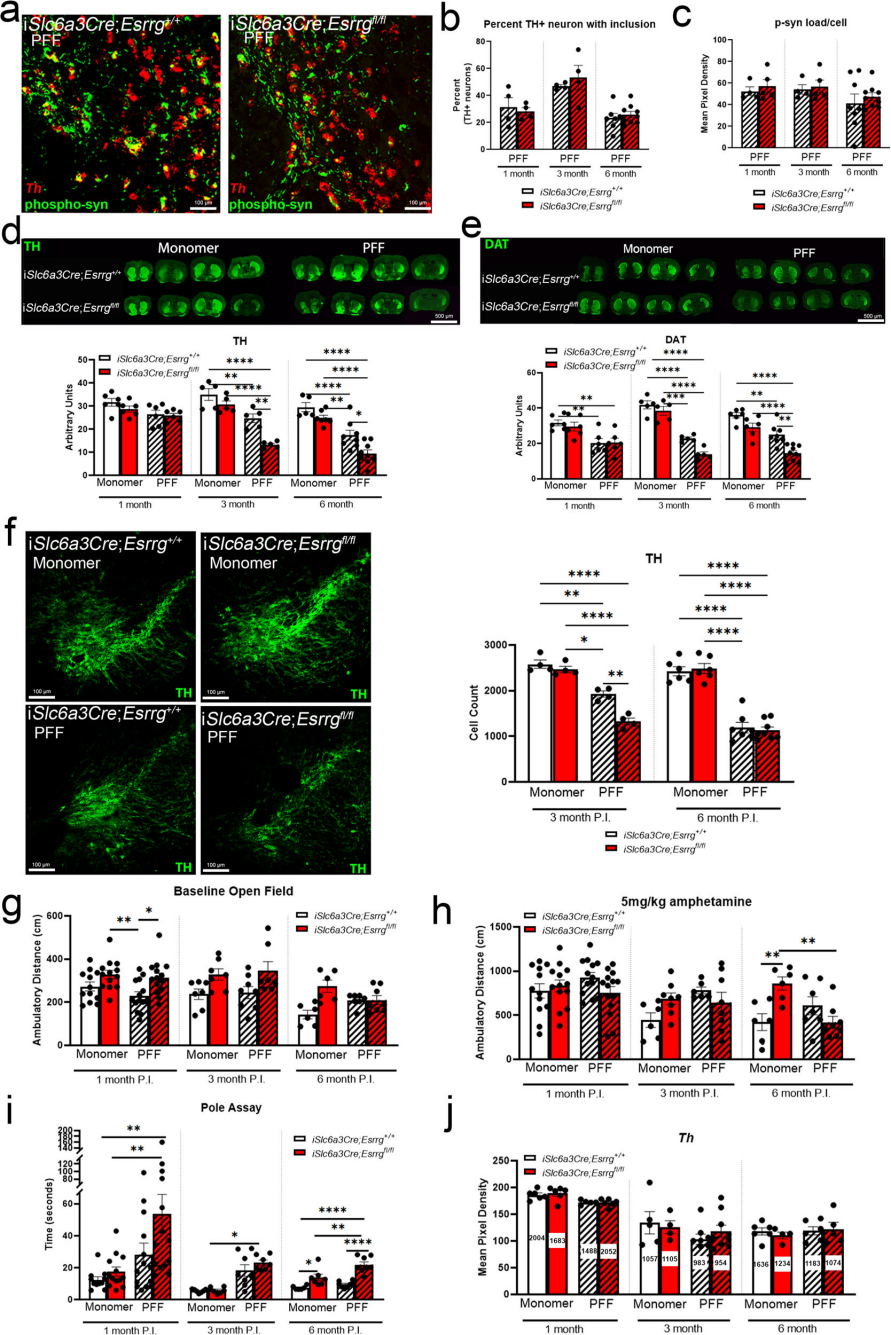
Partial deletion of *Esrrg* accelerates terminal and cell loss in the pre-formed fibril (PFF) model of synucleinopathy. **a** sm-FISH for *Th* (red) followed by immunofluorescence for phosphorylated α-synuclein (green). **b** Percent of *Th*+ neurons with an inclusion 1, 3 and 6 months post-injection (P.I.) of tamoxifen and PFFs (*n* = 5–9 mice/group; two-tailed unpaired *t*-test, n.s.). **c** Mean pixel density (occupancy of the cytoplasm) for phosphorylated α-synuclein (p-syn) per neuron 1, 3 and 6 months P.I. (*n* = 5–9 mice/group; two-tailed unpaired *t*-test at each time-point, n.s.). **d**, **e** TH or DAT immunofluorescence in the striatum at 3 months P.I. with quantification at 1, 3 and 6 months P.I. (*n* = 5–9/group, mixed-effects analysis with Sidak’s post hoc analysis at each time-point *p < 0.05, **p < 0.01,***p < 0.001, ****p < 0.0001). **f** Immunofluorescence for TH in the SNc 3 months P.I. (3 months: *n* = 5 mice/group, 6 months: *n* = 7–9/group; Mixed-effects analysis with Sidak’s post hoc analysis at each time-point *p < 0.05, ****p < 0.0001). **g**–**i** Ambulatory behavior and pole assay test 1, 3 and 6 months P.I. (*n* = 6–14 mice per group; Mixed-effects analysis with Sidak’s post hoc analysis at each time-point *p < 0.05, **p < 0.01, ***p < 0.001, ****p < 0.0001). **j** sm-FISH, single-cell *Th* mRNA analysis in all groups at all time-points P.I. (1 month: *n* = 6 mice/group, 3 months: *n* = 5/group, 6 months: *n* = 7–9/group; Mixed-effects analysis with Sidak’s post hoc analysis at each time-point, n.s.). Numbers on bars are cell counts from each experiment. Scale bars correspond to 100 µm (**a**, **f**), and 500 µm (**d**, **e**). Error bars represent ±SEM.

As any disease-related impairment in ERRγ activity or expression would not necessarily cause a complete loss of ERRγ as observed with the AAV-*ThCre* model, we sought to explore the effects of partial deletion of ERRγ on gene expression and behavior. We found that *Esrrg* abundance was reduced by ~60% in i*Slc6a3Cre*;*Esrrg*^*fl/fl*^ mice with respect to i*Slc6a3*Cre;*Esrrg*^*+/+*^ mice 1 month post-injection of tamoxifen; single-cell-level analyses revealed a reduction in *Esrrg* expression in tamoxifen-injected i*Slc6a3*Cre;*Esrrg*^*fl/fl*^ mice across all DAergic neurons in the SNc compared to control (Fig. 3a, b), although not to the extent observed with direct AAV-*ThCre* injection. There was no difference in *Esrrg* mean pixel density in the substantia nigra pars reticulata (SNr; Fig. 3c), demonstrating selectivity of deletion. At 4 months post-tamoxifen injection, hyperactivity in open field was observed and maintained until 9 months post-injection (Fig. 3d), with enhanced responsivity to AMPH as early as 4 months post-injection (Fig. 3e). Hyperactivity following AMPH administration was sustained until 9 months post-injection (Fig. 3e) at which point the observed pole assay deficit was the most robust (Fig. 3f). Reductions in *Atp5a1* and mitochondrial RNA (*mt-Cytb*) content were confirmed at 1 month post-tamoxifen injection in i*Slc6a3Cre*;*Esrrg*^*fl/fl*^ mice compared to control (Fig. 3g, h and Supplementary Fig. 4a, b). Distribution analyses reveal that the frequency of low *mt-Cytb* and *Atp5a1*-expressing cells is increased in the i*Slc6a3Cre*;*Esrrg*^*fl/fl*^ mice (Fig. 3f, g). Striatal TH immunoreactivity was intact at 9-month post-tamoxifen injection, suggesting lack of cell loss at that time point (Supplementary Fig. 4c and Quantitatively assessed in Fig. 4d–f).

Based on the above data, the reductions in putative ERRγ targets in DAergic neurons from patients with Lewy body pathology^12^, and prior evidence for synuclein-induced mitochondrial impairment^45,46^, we sought to explore several possibilities: (1) whether deficiency in ERRγ could render DAergic neurons more vulnerable to synuclein-mediated toxicity, (2) if overexpression of ERRγ could be neuroprotective in models of synucleinopathy, and (3) whether deficiencies in ERRγ-dependent transcription could be caused by synucleinopathy.

### Partial knockdown of *Esrrg* accelerates terminal and neuron loss associated with the preformed fibril model

To determine whether a reduction in *Esrrg* expression could influence vulnerability to alpha-synuclein pathology, we injected pre-formed fibrils (PFF) of alpha-synuclein into i*Slc6a3Cre;Esrrg*^*+/+*^ and i*Slc6a3Cre*;*Esrrg*^*fl/fl*^ striata and assessed neuron loss, motor behavior, and synuclein inclusion load. The PFF model of synucleinopathy was chosen due to its robust and measurable progression of DAergic terminal and cell loss over a 6-month period, mimicking the progression of DAergic cell loss in PD patients^47–52^. The *iSlc6a3Cre;Esrrg*^*fl/fl*^ line was chosen for these experiments because neither robust terminal or cell loss nor hypoactivity in open field was observed in this line (Supplementary Figs. 2c and 3c–e). Tamoxifen-induced recombination was initiated immediately prior to striatal PFF injections, to avoid any impact of *Esrrg* deletion on the initial uptake of PFFs into DAergic terminals. For all time points, open field and pole assay tests were performed and fresh-frozen tissues were collected to assess striatal TH and DAT immunoreactivity, single-cell mRNA measurements with sm-FISH in neurons with and without phosphorylated synuclein (p-syn), and approximation of DAergic cell number with NeuN, TH, cresyl violet, and DAPI labeling.

sm-FISH for *Th* with post-immunofluorescence for p-syn showed that the percent of *Th*+ neurons with inclusions and the area of the cytoplasm occupied by p-syn immunoreactivity was unchanged in i*Slc6a3*Cre;*Esrrg*^*fl/fl*^ as compared to i*Slc6a3Cre*;*Esrrg*^*+/+*^ mice at 1-, 3-, and 6-months (Fig. 4a–c). At 1 month post-injection, no differences were detected for TH staining intensity in the striatum; however, DAT was reduced in mice injected with PFF compared to monomer groups, regardless of genotype (Fig. 4d, e and Supplementary Fig. 4d). As reported previously in this model^47,48^, we replicate a reduction in striatal intensity for TH and DAT at 3 and 6 months post-injection, with PFF-injected i*Slc6a3Cre*;*Esrrg*^*fl/fl*^ mice exhibiting a significantly more severe reduction as compared to PFF-injected i*Slc6a3Cre*;*Esrrg*^*+/+*^ mice with no changes in the cortex or olfactory tubercle (Fig. 4d, e and Supplementary Fig. 2b, e). Regarding DAergic integrity in the SNc, we observed a reduction in the number of TH+ neurons in i*Slc6a3Cre*;*Esrrg*^*+/+*^ mice injected with PFFs as compared to mice injected with monomer at 3 and 6 months post-PFF injection (Fig. 4f and Supplementary Fig. 4e), with greater cell loss occurring at 3 months post-injection in i*Slc6a3Cre*;*Esrrg*^*fl/fl*^ mice (Fig. 4f). It is interesting to note that despite the similar extent of cell loss between PFF groups at 6 months, TH and DAT terminal density is even further reduced in the i*Slc6a3Cre*;*Esrrg*^*fl/fl*^ mice, suggesting a dependence on *Esrrg* for maintenance of DAergic terminals of remaining neurons (Fig. 4d–f and Supplementary Fig. 4d). Importantly, there were no differences in TH or DAT immunoreactivity, or cell counts with deletion of *Esrrg* in the monomer group, indicating that partial *Esrrg* deficiency is not sufficient to affect DAergic terminals or cell bodies and that a synergistic relationship between *Esrrg* deletion and PFF treatment exists regarding synaptic and cellular toxicity.

To understand motor phenotypes associated with ERRγ-loss and the PFF model, we assessed baseline ambulatory activity and coordination using open field followed by administration of AMPH to unmask any underlying dysfunction in the nigral-striatal pathway^53–55^. Baseline open field at 1 month post-injection showed that mice from both i*Slc6a3Cre*;*Esrrg*^*fl/fl*^ groups, regardless of treatment, were hyperactive compared to the i*Slc6a3Cre*;*Esrrg*^*+/+*^ mice injected with PFFs (Fig. 4g). Behavioral observations 6-months post-injection showed that i*Slc6a3Cre*;*Esrrg*^*fl/fl*^ mice injected with monomer were hyperactive compared to i*Slc6a3Cre*;*Esrrg*^*+/+*^ mice injected with monomer (Fig. 4g). When mice were injected with 5 mg/kg of AMPH, there were no differences across treatment groups at the 1 or 3-month time points (Fig. 4h). By 6 months post-injection, the i*Slc6a3Cre*;*Esrrg*^*fl/fl*^ mice injected with monomer had a hyperactive response to AMPH as compared to i*Slc6a3Cre*;*Esrrg*^*+/+*^ mice injected with monomer as well as the i*Slc6a3Cre*;*Esrrg*^*fl/fl*^ mice injected with PFFs (Fig. 4h). This observation could be due to the loss of terminals associated with ERRγ deletion and injection of PFFs which is worse than with wild-type mice injected with PFFs. This idea is further corroborated by the pole assay where i*Slc6a3*Cre;*Esrrg*^*fl/fl*^ mice injected with PFFs perform worse on the pole assay task at both 1 and 6 months post-injection as compared to mice injected with monomer (Fig. 4i). Interestingly, by 6 months post-injection, i*Slc6a3Cre*;*Esrrg*^*fl/fl*^ mice injected with monomer perform worse as compared to i*Slc6a3Cre*;*Esrrg*^*+/+*^ injected with monomer (Fig. 4i), similar to the pole assay deficits seen in non-PFF-injected i*Slc6a3Cre*;*Esrrg*^*fl/fl*^ mice (Fig. 3e). Importantly, we found that *Th* mRNA expression per cell was not affected at any time point, in any group, indicating that reductions in TH protein or TH+ cell counts were not due to effects on *Th* transcription (Fig. 4j). Altogether, these data suggest that ERRγ deficiency and PFF-mediated toxic processes synergistically influence DAergic vulnerability.

### Overexpression of *Esrrg* in dopaminergic neurons causes an upregulation of mitochondrial genes and protects dopaminergic terminals in the preformed fibril model

**Fig. 5:**
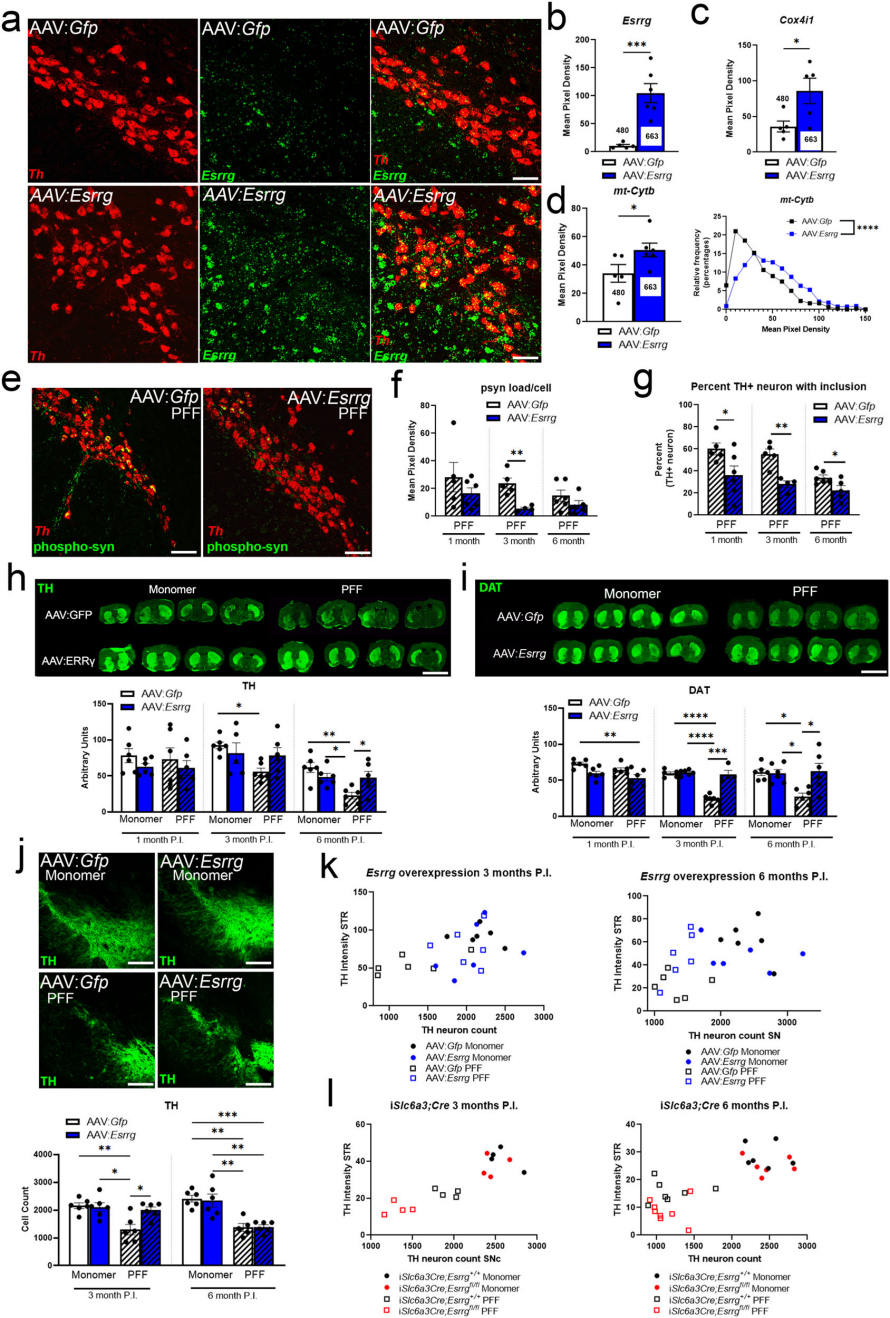
Overexpression of *Esrrg* in the midbrain causes an induction in mitochondrial genes and delays cell death in the PFF model of synucleinopathy. **a** sm-FISH for *Th* (red) and *Esrrg* (green) in mice with AAV:*Gfp* or AAV:*Esrrg* midbrain injections, quantified in **b** (*n* = 6 mice/group; two-tailed unpaired *t*-test *p < 0.05). **c**, **d** sm-FISH quantification for nuclear encoded mitochondrial gene *Cox4i1* and mitochondrially encoded gene *mt-cytb* (*n* = 6/group two-tailed unpaired *t*-test or unpaired nonparametric Kolmogorov–Smirnov test ****p < 0.0001). **e** sm-FISH for *Th* (red) followed by immunofluorescence for phosphorylated α-synuclein (p-syn; green) at 1 month post-injection (P.I.). **f** Mean pixel density (occupancy of the cytoplasm) for p-syn per neuron at 1, 3 and 6 months P.I. (*n* = 4–6 mice/group; two-tailed unpaired *t*-test at each time-point **p < 0.005). **g** Percent of *Th*+ with presence of an inclusion for p-syn (*n* = 4–6 mice/group; two-tailed unpaired *t*-test at each time-point *p < 0.05, **p < 0.005). **h**, **i** Immunofluorescence for TH or DAT in the striatum of mice injected with AAV:*Gfp* or AAV:*Esrrg* and/or monomer or PFFs (*n* = 6 mice/group; mixed-effects analysis with Sidak’s post hoc analysis at each time-point *p < 0.05, **p < 0.005, ***p < 0.001, ****p < 0.0001). **j** Neurons positive for TH immunoreactivity in mice injected with AAV:*Gfp* or AAV:*Esrrg* and/or monomer or PFFs (*n* = 6 mice/group; mixed-effects analysis with Sidak’s post hoc analysis at each time-point *p < 0.05, **p < 0.05). **k**, **l** Scatter plots to graph SNc TH neuron count and striatal TH intensity per animal at 3 and 6-months P.I. Numbers on bars are cell counts from each experiment. Scale bars correspond to 50 µm (**a**),100 µm (**e**, **j**), and 500 µm (**h**, **i**). Error bars represent ±SEM.

We next evaluated whether ERRγ overexpression is neuroprotective in the PFF model. Using ERRγ as a neuroprotective strategy could be particularly relevant for PD therapeutics, as ERRγ agonists have been identified^36,56–58^. We designed an AAV to overexpress *Esrrg* in the midbrain (AAV5:CAG-*Esrrg-*IRES-EGFP-WPRE). We first confirmed overexpression of *Esrrg* mRNA in the midbrain 1 month post-injection via sm-FISH (Fig. 5a, b) and found that *Esrrg* overexpression increased the expression of *Cox4i1* as well as *mt-Cytb* per DAergic neuron (Fig. 5c, d and Supplementary Fig. 5a, b). Wildtype mice were then injected concurrently with AAV-*Gfp* (control; AAV5:*Gfp*) or AAV-*Esrrg* bilaterally into the SNc and monomer or PFFs of α-synuclein bilaterally into the dorsolateral striatum. All assessments performed for *Esrrg*-deficient mice (Fig. 4) were performed for these cohorts. In contrast to PFF-injected mice with *Esrrg* knockdown for which no alterations were observed with inclusion load (Fig. 4b, c), mice receiving AAV-*Esrrg* injections exhibited a reduction in the proportion of DAergic neurons bearing an inclusion and the area of the cell occupied by p-syn (Fig. 5e–g). Remarkably, while reductions in striatal TH and DAT immunoreactivity occurred with age in AAV-*Gfp*;PFF-injected mice with no changes in the cortex or olfactory tubercle (Supplementary Fig. 2c, f), no TH or DAT reductions were observed in mice receiving AAV-*Esrrg* injections at any time point (Fig. 5h, I and Supplementary Fig. 5c). At 3-months, the AAV-*Esrrg* PFF group had no detection of cell loss (Fig. 5j and Supplementary Fig. 5d); however, despite the maintenance of TH and DAT immunoreactivity in the striatum, the reduction in cell counts was just as pronounced in the AAV-*Esrrg* PFF group as in the AAV-*Gfp* PFF group at 6 months, suggesting remodeling of existing neurons in the presence of ERRγ- overexpression (Fig. 5j). In light of the disconnect between the cell count and TH/DAT intensity in the 6 month AAV-*Esrrg* PFF mice, we sought to confirm maintenance of striatal TH abundance with western blotting (Supplementary Fig. 6). Western blotting results mirror immunofluorescence with a loss of TH in AAV-*Gfp*;PFF-injected mice but not in the AAV-*Esrrg* PFF group. No behavioral differences were noted in baseline or AMPH-induced ambulatory activity or performance in the pole assay (Supplementary Fig. 5e–g), except for a modest reduction in AMPH-induced locomotion with *Esrrg* overexpression in the monomer group at 6 months post-injection (Supplementary Fig. 5f). *Th* mRNA/cell was also unchanged in all groups (Supplementary Fig. 5h). To better visualize the relationship between striatal innervation and cell count in individual mice in the knockdown and overexpression models, we plotted cell count with respect to TH immunoreactivity (Fig. 5k, l). With these comparisons, it is clear that ERRγ content is positively associated with the maintenance of DAergic terminals, with overexpression supporting maintenance of terminals despite loss of DAergic cells (Fig. 5k, l).

### Modulation of ERRγ causes changes in predicted ERRγ-dependent genes

**Fig. 6:**
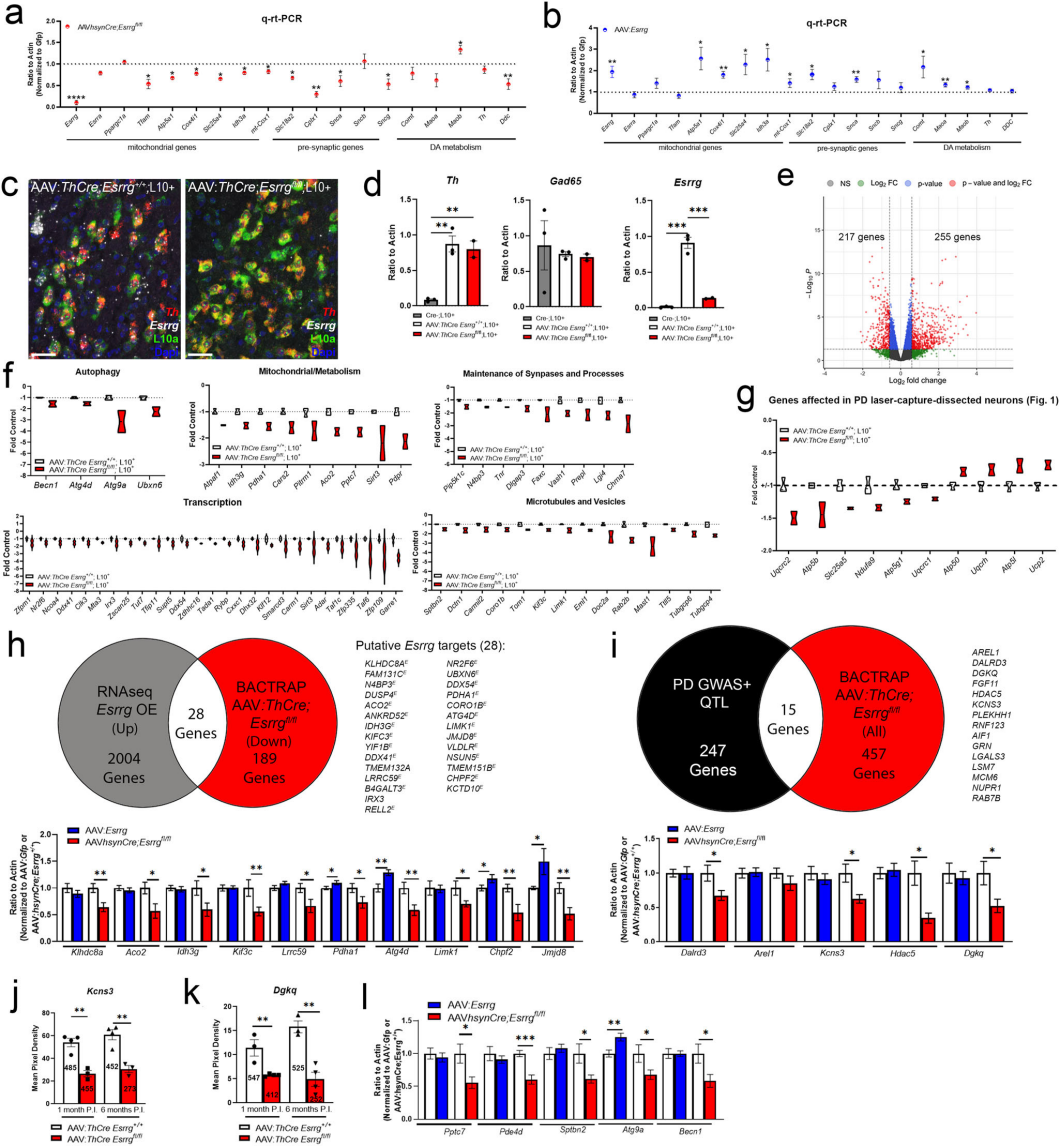
BAC-TRAP from mice lacking *Esrrg* in DAergic neurons revealed genes related to autophagy, mitochondrial and synaptic function, transcription, and microtubule- and vesicle-related pathways. **a**, **b** q-rt-PCR data from midbrain of mice lacking *Esrrg* (AAV-*hsynCre Esrrg*^*fl/fl*^ vs. AAV-*hsynCre Esrrg*^*+/+*^) or of mice overexpressing *Esrrg* (AAV-*Esrrg* vs. AAV-*Gfp*) (*n* = 7–8/group; two-tailed unpaired *t*-test *p < 0.05, **p < 0.01). **c** Representative images from sm-FISH for *Th* (red), *Esrrg* (white), endogenous GFP-L10a (green), and DAPI (blue) in *Esrrg*^*+/+*^;L10+ and *Esrrg*^*fl/fl*^; L10+ mice injected with AAV:*ThCre* to induce the *Gfp-Rpl10a* transgene in DAergic neurons. **d** q-rt-PCR from BAC-TRAP pulldowns for *Th*, *Gad65* and *Esrrg* transcript for confirmation of enrichment of DAergic markers and exclusion of inhibitory neuron markers (*n* = 2–3/group; one-way ANOVA with Tukey’s post hoc analysis **p < 0.01, ***p < 0.001). **e** Volcano plot showing differentially expressed genes in AAV:*ThCre*-injected *Esrrg*^*+/+*^*;*L10+ and *Esrrg*^*fl/fl*^; L10+ mice after sequencing (gray = no significance, green = ±1.5 log_2_-fold change, blue = significant *p*^adj^ value, red = significant *p*^adj^ value and differentially expressed ±1.5 log_2_-fold change). **f** Fold control expression of genes downregulated with *Esrrg* deletion by functional category. **g** Fold control of ETC genes reduced in PD patients that had significant *p*^adj^ values but not ±1.5 log_2_ fold change. **h** Pie chart demonstrating overlap between genes changed with *Esrrg* overexpression in SH-SY5Ys and genes changed with *Esrrg* knockout with BAC-TRAP. Q-rt-PCR with *Esrrg* knockout or overexpression from genes identified as putative targets of *Esrrg* (*n* = 5–8 mice/group; two-tailed unpaired *t*-tests *p < 0.05, **p < 0.01). **i** Overlap of predicted PD GWAS and QTL genes and genes changed with *Esrrg* knockout using BAC-TRAP in DAergic neurons. qPCR from targets in both *Esrrg* knockout and overexpression in the midbrain (*n* = 5–8 mice; two-tailed unpaired *t*-test *p < 0.05). **j**, **k** sm-FISH for the identified targets *Kcns3* and *Dgkq* at both 1 and 6 months P.I. of AAV-*ThCre* (*n* = 3–4/group; two-tailed unpaired *t*-test *p < 0.05, **p < 0.01). **l** q-rt-PCR from *Esrrg* knockout (AAV-*hsynCre*) or overexpression (AAV-*Esrrg*) midbrain homogenate for autophagy and microtubule and vesicle-related genes (*n* = 5–8 mice/group; two-tailed unpaired *t*-test *p < 0.05, **p < 0.01, ***p < 0.001). Numbers on bars are cell counts from each experimental group. Scale bars correspond to 50 µm (**c**). Error bars re*p*resent ± SEM.

To explore the gene changes underlying these observed phenotypes, we used several approaches, including the measurement of predicted ERRγ-dependent genes using rt-PCR in *Esrrg*-deficient and *Esrrg*-overexpressing midbrain homogenates (Fig. 6a, b) and the unbiased profiling of *Esrrg*-deficient DAergic neurons using translating ribosome affinity purification and RNA-seq (Fig. 6c–g). Mice lacking *Esrrg* expression in midbrain neurons showed deficiencies in the known *Esrrg* targets (Pei et al.^26^) *Atp5a1, Cox4i1, Slc25a4*, and *Idh3a* (Fig. 6a) in the absence of significant changes in mRNA levels for *Ppargc1a*, *Esrra*, or *mt-Cox1* (Fig. 6a). *Tfam*, the gene known to control expression of mitochondrially-encoded genes, was also reduced (Fig. 6a). We also found reductions in pre-synaptic genes dependent on PGC-1α, including *Cplx1*, *Slc18a2* (encoding VMAT2), *Sncg*, and the DA metabolizing enzyme *Ddc* (Fig. 6a). In contrast, most, but not all, ERRγ-dependent and -independent genes tested were increased with *Esrrg* overexpression (*Atp5a1*, *Cox4i1*, *Slc25a4*, *Idh3a*, *mt-Cox1*, *Slc18a2*, *Snca*, *Maoa*, and *Maob*).

### BAC-TRAP from mice lacking *Esrrg* in dopaminergic neurons reveals ERRγ-dependent genes

Considering this robust transcriptional response, we explored the *Esrrg*-dependent transcriptome in an unbiased manner using bacterial artificial chromosome translational affinity purification (BAC-TRAP). This method uses Cre-dependent expression of EGFP-tagged ribosomal protein Rpl10a (L10) to enable the immunoprecipitation of transcripts from discrete neuronal cellular populations in vivo^59,60^. Samples were generated by backcrossing the L10 mouse onto the *Esrrg*^fl/fl^ background to generate *Esrrg*^+/+^;L10^+^ and *Esrrg*^fl/fl^;L10^+^ mice, which were then injected with AAV-*ThCre* into the midbrain to activate the transgene selectively in DAergic neurons. RNA was isolated at 1 month post-injection to identify transcriptional changes that precede cell dysfunction and death. Enrichment of DAergic transcripts was confirmed prior to RNA sequencing (Fig. 6d); samples from *Esrrg*^+/+^;L10^+^ and *Esrrg*^fl/fl^;L10^+^ mice showed enrichment of *Th* expression with no enrichment in *Gad65* expression as compared to Cre-negative mice, demonstrating specificity for DAergic neurons (Fig. 6d). *Esrrg* was also significantly reduced in *Esrrg*^fl/fl^ samples compared to *Esrrg*^+/+^ samples in this cohort (Fig. 6d), and splicing analyses confirmed the deletion of *Esrrg* exon 2 in these samples from *Esrrg* transcript variant 1 (Nm_011935.3; Supplementary Fig. 4a). RNA sequencing identified an upregulation of 255 genes and downregulation in 217 genes (±1.5-fold, *p*adj < 0.05; Fig. 6e). GO analysis using Webgestalt and Enrichr in conjunction with PubMed revealed downregulated genes in autophagy, mitochondrial and metabolic function, maintenance of synapses and neuronal processes, transcription, and vesicle-mediated transport (Fig. 6f). Upregulated genes were associated with immune response, transcription, and the cytoskeleton as well as ribosomal support (Supplementary Fig. 7b). Interestingly, when we cross-referenced our BAC-TRAP data with the list of mitochondrial genes reduced in laser-captured neurons from patients^12^ (Fig. 1a) and filtered for genes that were significant (*p*adj < 0.05) but not ±1.5-fold in difference, we did find differences in 10 genes (*Uqcrc2, Atp5b, Slc25a5, Ndufa9, Uqcrc1, Atp5o, Uqcrh, Atp5l*, and *Ucp2*; Fig. 6g). These data suggest that deletion of *Esrrg* is sufficient to cause the dysregulation of mitochondrial genes associated with PD.

Subsequently, we prioritized genes for in vivo validation using several criteria: downregulation of the gene in BAC-TRAP data (more likely than upregulated genes to be direct targets of ERRγ transcriptional activation programs), demonstration of regulation by ERRγ overexpression in the catecholaminergic SH-SY5Y cell line, overlap with genes implicated by PD GWAS and QTL studies, and expression in DAergic neurons of the mouse brain (Supplementary Fig. 7c; Dropviz.org^30^). We found that 67/2525 of the genes significantly altered with ERRγ-overexpression in SY5Ys were also reduced in the BAC-TRAP dataset (Fig. 6h). Interestingly, of these genes, 28 were increased with ERRγ-overexpression while 39 were reduced, suggesting that the presence of ERRγ can be associated with either transcriptional coactivation or repression, depending on the cellular context (Fig. 6h). Of the 28 genes upregulated with ERRγ overexpression we chose *Kldhc8a, Aco2, Idh3g, Kif3c, Lrrc59, Pdha1, Atg4d, Limk1*, *Chpf2*, and *Jmjd8* to validate using qPCR from mRNA from mice with ERRγ-knockout or overexpression in the midbrain, based on their expression in mouse DAergic neurons (Supplementary Fig. 7c). While we found a reduction in *Kldhc8a, Aco2, Idh3g, Kif3c, Lrrc59 and Limk1* with *Esrrg* knockdown, not all were upregulated with ERRγ-overexpression, except for the autophagy related gene *Atg4d*, a nuclear-encoded mitochondrial multienzyme complex gene *Pdha1*, Chondroitin Polymerizing Factor 2 (*Chpf2*) and a gene related to Golgi function, *Jmjd8*. (Fig. 6h). Of note, using the UCSC genome browser, we determined that 26/28 genes show evidence of being bound by ERRα by chromatin immunoprecipitation (as noted with a superscript “E” in Fig. 6h and Supplementary Table 2), suggesting the possibility for direct interaction of ERR family members with those genes.

When the genes downregulated in the BAC-TRAP dataset were compared to genes implicated by PD GWAS and QTL (see methods), 15 genes were found; four neuron-enriched genes were chosen for confirmation; *Dalrd3*, *Kcns3*, *Hdac5*, and *Dgkq* were reduced with knockdown and not increased with overexpression (Fig. 6i). Considering the possibility that the rt-PCR results could be reflecting changes in local GABAergic neurons in homogenates, we confirmed the reduction of two genes, *Kcns3* and *Dgkq*, in DAergic neurons using sm-FISH (Fig. 6j, k and Supplementary Fig. 7d).

To further explore genes which may be involved in reduction in p-syn pathology and maintenance of DAergic terminals with *Esrrg* overexpression, we chose several other BAC-TRAP downregulated genes related to autophagy, mitochondrial function, and synaptic remodeling. We found a reduction in the autophagy genes, *Atg9a*, and *Becn1* with *Esrrg* deficiency and an increase in *Atg9a* with overexpression (Fig. 6l). We also found reductions in the synaptic remodeling gene *Sptbn2* with *Esrrg* knockdown that did not change with overexpression, similar to the mitochondrial gene *Pptc7* and the cAMP-hydrolyzing enzyme in cells *Pde4d* (Fig. 6l). Interestingly, mice lacking *Esrrg* in forebrain glutamatergic neurons, as a comparison to DAergic neurons (Supplementary Fig. 8), show deficiencies in a subset of non-mitochondrial genes including *Atg4d* and *Atg9a* but few changes in ERRγ-dependent mitochondrial genes in the hippocampus, suggesting cell type-specific requirements for *Esrrg* in gene expression (Supplementary Fig. 8b, c).

### Synucleinopathy is sufficient to alter a subset of ERRγ-dependent genes

**Fig. 7:**
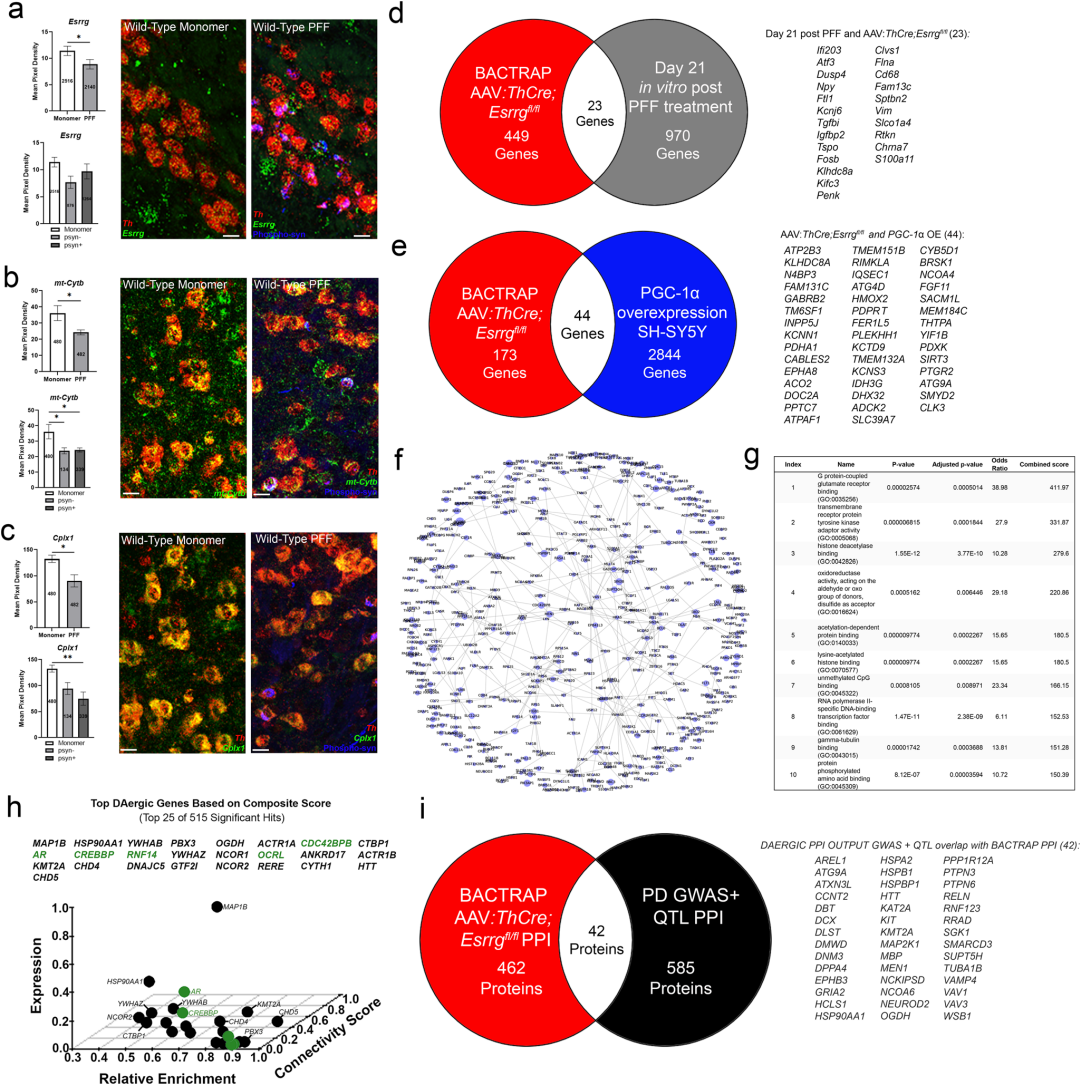
*Esrrg*-dependent pathway disruption in PFF-containing neurons and other PD models. **a**–**c** sm-FISH quantification of select *Esrrg*-dependent genes in DAergic neurons with and without phosphorylated α-synuclein (p-syn) inclusions (*n* = 6–14/group; two-tailed unpaired *t*-test or one-way ANOVA with Tukey’s post hoc analyses *p < 0.05, **p < 0.01). **d** Venn diagram showing overlap with genes from BAC-TRAP with *Esrrg* deletion and day 21 post in vitro PFF treated neurons. **e** Venn diagram showing overlap between BAC-TRAP with *Esrrg* deletion and PGC-1α overexpression in SH-SY5Y’s. **f** Protein-protein-interaction model to identify convergent targets of transcripts altered by *Esrrg* deletion as detected by BAC-TRAP. **g** Gene Ontology molecular function as identified with Enrichr from proteins generated from BAC-TRAP PPI. **h** Top DAergic neuron hits from BAC-TRAP PPI data by connectivity score and abundance and relative enrichment in DAergic neurons; green signifies druggable target. **i** Overlap of BAC-TRAP PPI proteins with convergent proteins identified in a PPI generated from PD GWAS and QTL. Numbers on bars are cell counts from each experiment. Scale bars correspond to 10 µm (**a**–**c**). Error bars represent ±SEM.

Considering the reduction in mitochondrial gene expression in patients with synucleinopathy, we questioned whether synucleinopathy is sufficient to alter expression of a subset of the ERRγ-dependent genes in wild-type DAergic neurons injected with PFFs. Using FISH, we found reductions in *Esrrg*, *mt-Cytb* and *Cplx1* in DAergic neurons from mice injected with PFFs (Fig. 7a–c), with no changes in *Dgkq*, *Cox4i1*, and *Atp5a1* (Supplementary Fig. 9a–c). Interestingly, there was no difference in *Esrrg*, *mtCytb*, or *Cplx1* expression between neurons with or without inclusions (Fig. 7a–c), suggesting that transcriptional differences may not be caused by the formation of an inclusion itself (Fig. 7a–c). To further investigate this idea, we cross-referenced our BAC-TRAP list to a list of genes that changed at day 1, 3, 7, 14 and 21 from cortical hippocampal neurons exposed to PFFs in culture. We found a total overlap of 28/1239 genes with the most overlap between day 21 and genes that change with *Esrrg* deletion with an overlap of 23/1016 genes (Fig. 7d and Supplementary Fig. 9d). Significantly, 3 of the overlapped genes (*Kldhdc8a*, *Kif3c* and *Sptbn2*) were also reduced in homogenates from *Esrrg-*deficient mice, and *Kif3c* and *Sptbn2* are important for microtubule and vesicle function (Fig. 6m). From these data, we conclude that synucleinopathy has the potential to disrupt transcriptional programs for mitochondrial and synaptic function that could impact DAergic vulnerability, potentially explaining the synergistic interaction between PFFs and ERRγ deficiency.

### ERRγ regulates similar genes as PGC-1α

Considering previous evidence for the neuroprotective efficacy of PGC-1α overexpression in some PD models^12,18,23,61^, we cross-referenced genes altered in *Esrrg*-deficient DAergic neurons with genes changed with PGC-1α overexpression in SH-SY5Y’s^29^. We found that, of the genes upregulated with PGC-1α overexpression and genes downregulated with *Esrrg* deletion, there was an overlap of 44 genes (Fig. 7e). Interestingly, the PD associated gene *Kcns3*, as well as *Pptc7*, *Kldhdc8a*, and autophagy genes *Atg4d* and *Atg9a* were all found to overlap between the two lists and were changed in *Esrrg*-deficient mice (Fig. 6h, i, l).

### Models demonstrate the overlap between ERRγ-deficient dopaminergic neurons and neurons affected in Parkinson’s disease

To investigate further the possible functional similarities between *Esrrg*-deficient DAergic neurons and DAergic neurons from PD patients, we generated a model to predict what downstream processes could be affected by changes in the differentially expressed genes. To do this, we generated a PPI network using a combination of BAC-TRAP data and BioGRID protein-protein-interaction data to identify genes/proteins which could be convergent downstream targets of the genes up-and down-regulated by *Esrrg* deficiency (Fig. 7f). Importantly, prior to generating the model, we removed all genes that are not expressed in mouse DAergic neurons from the BioGRID list (Fig. 7f; cross-reference with Dropviz.org^30^). GO analyses of significantly enriched proteins in the model revealed convergence on G protein-coupled glutamate receptor binding, transmembrane receptor protein tyrosine kinase adapter activity, and histone deacetylase binding (Fig. 7g). Top hits were identified by taking into consideration connectivity values within the model, abundance of expression, and relative enrichment of expression in DAergic neurons (Fig. 7h; see methods).

A similar process was performed using DAergic neuron-enriched genes associated with PD risk to generate models of DAergic neuron dysfunction in idiopathic PD (see methods). There was an overlap of 42 proteins between the two lists (Fig. 7i). From this overlap, 8 proteins were also found on the PD GWAS + QTL list including AREL1, CCNT2, DLST, DNM3, KAT2A, NCKIPSD, RNF123, and VAMP4 (Fig. 7i). One autophagy protein, ATG9A, was also found in mice deficient in *Esrrg* through BAC-TRAP (Fig. 6f) and was confirmed as reduced with qPCR with knockout and increased with *Esrrg* overexpression (Figs. 6l and 7i). This overlap suggests that DAergic neurons deficient in *Esrrg* and neurons dysfunctional in PD are similar regarding the genes/proteins necessary for proper DAergic neuronal function.

## Discussion

Mitochondrial dysfunction has been extensively studied in its relation to PD; however, the mechanism underlying a deficiency in nuclear-encoded mitochondrial gene expression is unknown. PGC-1α is a proposed upstream regulator of nuclear-encoded genes that are deficient in PD^12,14^; however, PGC-1α knockout mice do not show overt loss of DAergic terminals or cell bodies^16^, suggesting that other factors are involved in the regulation of these genes in normal states. ERRγ is a transcription factor known to regulate mitochondrial genes^24–26,32,34^ and, here, we document its expression in nigral-striatal DAergic neurons and show DAergic dependence on ERRγ for maintenance of gene expression and viability with age. Viral-mediated knockdown of *Esrrg* selectively in adult DAergic neurons caused synaptic and somatic loss, a reduction in mitochondria per cell cross-section, and an L-DOPA-responsive impairment in ambulation and coordination. Important for our understanding of ERRγ’s potential role in PD etiology, we did not see a large overlap in genes reduced with ERRγ deletion and those reduced in postmortem tissue from patients with synucleinopathy. These data indicate that while ERRγ is necessary for maintenance of normal DAergic viability and gene expression, it is unlikely that a reduction in ERRγ abundance (or activity) is solely responsible for the transcriptional deficiencies observed in PD.

DAergic neurons are particularly vulnerable to oxidative stress and mitochondrial dysfunction^3–9^, as demonstrated by several in vivo studies using toxins like MPTP, rotenone, and 6-OHDA^62^. A recent study demonstrates that the deletion of *Ndufs4* from DAergic neurons can create a PD-like phenotype in mice by shifting from a dependence on oxidative phosphorylation to glycolysis^10^. Other evidence stems from genetic studies which implicate mitochondrial function in PD risk link (loss-of-function mutations in *PARK2*/Parkin^63^, *PARK6*/PINK1^63,64^ and *PARK7*/DJ-1^65^). We show that ERRγ deletion causes a reduction in the number of mitochondria per cell cross-section as well as reduction in genes known to be regulated by mitochondrial transcription factor a (*Tfam*; Fig. 2). Interestingly, deletion of *Tfam* selectively in DAergic neurons (the “MitoPark” model) has been touted as a model for PD, showing a robust phenotype of mitochondrial dysfunction, neuron loss and DAergic neuron-specific motor phenotypes associated with a loss of *Tfam*-dependent mitochondrially-encoded genes^66^. Here, we are intervening upstream of *Tfam*, which could explain our reduction in both mitochondrially encoded genes as well as the reduction in number of mitochondria. Despite these similarities, we argue that the partial deletion of *Esrrg* may be a better tool for understanding DAergic vulnerability in PD, considering that shifts in transcriptional programs may be more likely to occur downstream of cellular signaling events in disease (see Fig. 7) and aging rather than isolated effects on *Tfam* alone. To investigate this idea further we generated a model to understand the functional similarities between *Esrrg*-deficient DAergic neurons and DAergic neurons from PD patients (Fig. 7). We found it interesting that both neurons deficient in *Esrrg* and DAergic neuron networks of PD risk genes were convergent on pathways related to synaptic and microtubule function, consistent with the emerging theories of early synaptic dysfunction in PD^67^.

However, it is important to note that other neuronal populations could be vulnerable to oxidative stress and/or the deletion of mitochondrial genes, an issue which is often not thoroughly explored in PD models. Certainly, there is abundant evidence for involvement of other cell types in the pathogenesis of PD, including glial cells^68–71^. Regarding the cellular specificity of ERRγ’s actions, we (Supplementary Fig. 8a, b), and others have shown that glutamatergic neuronal populations that lack ERRγ in the hippocampus show only mild functional deficits, even with reported changes in mitochondrial respiration^26^. These findings suggest that ERRγ’s role in DAergic neurons may be unique, although additional studies are required to define ERRγ’s roles in other cell types. Similar to what we observe with *Esrrg* deletion in DAergic neurons, genes encoding potassium channels are also downregulated in the heart with *Esrra/Esrrg* knockdown^32^. *Esrrg* transcript expression is also enriched in GABAergic neuron populations with high expression of the calcium buffer parvalbumin^29,30^, similar to the cell type-specific expression pattern of *Ppargc1a*; it would be interesting to determine the transcriptional and functional consequences of *Esrrg* deletion in those cells.

Gene changes in DAergic neurons from patients with presence of Lewy-body pathology upon post- mortem observation show reduction in nuclear-encoded genes of the ETC^12^. ChIP from *Esrrg*^26^-deficient fibroblasts showed overlap with this gene list leading us to hypothesize that *Esrrg* deletion would cause changes in genes similar to ones reduced in DAergic neurons in PD. We found that 5 of the 75 genes reduced in ref. ^12^ were also reduced in DAergic neurons lacking *Esrrg*, raising the possibility that other transcription factors could be maintaining the expression of the remaining genes. Other members of the ERR family (ERRα and ERRβ) can bind the same consensus binding site as ERRγ, meaning ERRγ target genes could be regulated by any of the 3 transcription factors^31,72^ depending on the cell type and stage of development (ERRα in adulthood, ERRβ in development)^72^. Interestingly, we found that *Esrra* expression was unchanged in mice lacking *Esrrg*; it is possible that ERRα is capable of maintaining subsets of ERRγ target genes. However, recently published data from *Esrra* knockout mice midbrain homogenate show no changes in metabolic transcripts *Atp5o*, *Atp5a1*, or *Idh3a*^29^. Considering that ERRα and ERRγ can form functional heterodimers^31^, it is possible that deletion of both factors would be required to see a widespread reduction in nuclear-encoded mitochondrial genes.

*Esrrg* modulation causes different phenotypes at the synapse depending on the direction of expression, with deletion causing reduction in terminals and overexpression providing protection against PFF-mediated terminal loss. Our BAC-TRAP data shows that ERRγ-dependent genes fall in categories for synaptic remodeling and microtubule function (*Sptbn2*, *Limk1* and *Kif3c*; Fig. 6), which could contribute to the observed phenotypes. However, none of the validated genes were also increased with overexpression, except for the genes in the autophagy category (*Atg4d, Atg9a)*, a metabolism-related gene (*Pdha1*), and a Golgi-related gene *Jmjd8*. These data are particularly interesting considering that both the percent of *Th*+ neurons with inclusions and the area of the cytoplasm occupied by p-syn immunoreactivity was reduced with *Esrrg* overexpression (Fig. 5). Several groups have demonstrated that inhibition of autophagy increases aggregated alpha-synuclein levels^73–75^ and, conversely, activation of autophagy specifically thorough Beclin 1 (*Becn1*) enhances the degradation of alpha-synuclein aggregated species^76^. Altogether, these findings suggest that a number of functional pathways are engaged by ERRγ overexpression; future studies are needed to explore whether individual functional pathways are the primary drivers of the neuroprotective response.

Strategies targeting mitochondrial function have been under consideration for the treatment of neurological disorders, with limited success in clinical trials (reviewed in refs. ^77,78^). Our findings of the neuroprotective effects of ERRγ overexpression raise the possibility that approaches which target multiple functional pathways could be more effective than strategies which target only mitochondria. A recent study demonstrated that a newly synthesized small molecule that binds and activates ERRγ, HPB2, stimulates BDNF/TrkB signaling both in vitro and in vivo, with upregulation in both TH and DAT expression^37^. Likewise, they demonstrated that the agonist for ERRγ, GSK4716, increases DAT and TH expression in differentiated SH-SY5Y neuroblastoma cells^36^. Conversely, the inverse agonist for ERRγ, GSK5182, attenuated the upregulation of DAT/TH, potentially via the cAMP/PKA/CREB protein signaling pathway^36^. It is important to note that we did not observe any changes in TH or DAT expression with *Esrrg* overexpression, suggesting that activation of ERRγ-responsive gene programs with an agonist versus ERRγ overexpression itself could elicit different effects. This could be explained by the possible engagement of transcriptional repressors with the overexpression of ERRγ, which may not necessarily be observed with an ERRγ agonist. In support of this idea, more genes were increased with ERRγ deletion than were reduced, indicating that ERRγ may work with repressors or coactivators to control gene expression, depending on the locus. In fact, previous studies have demonstrated that ERRγ can bind to both repressors and coactivators (reviewed in ref. ^79^). Also, it is important to note that transcriptional regulators like HDAC5 were reduced with ERRγ knockdown, which could lead to de-repression of a number of downstream transcripts that are not necessarily direct targets of ERRγ. Future experiments should explore the differences in the transcriptional responses to ERRγ agonism and ERRγ overexpression in vivo and whether existing ERRγ agonists are capable of crossing the blood brain barrier and activating ERRγ-dependent genes in vivo, without the detrimental effects sometimes observed with sustained, robust overexpression^80,81^.

Consistent with recent publications in primary neuronal culture^49,82–87^, we show an effect on transcription with the presence of pathological synuclein in vivo. The most comprehensive of these studies comes from Mahul-Mellier et al. 2019^85^ where researchers use a time-course study along with RNAseq to understand what genes are changing over time once hippocampal neurons are exposed to pathological synuclein using the PFF model. These studies were done in hippocampal neuronal culture, and we acknowledge that more robust changes could be revealed if similar studies were conducted in vivo specifically in DAergic neurons. The upstream processes responsible for these transcriptional changes are not clear. A number of pathological signaling processes could converge on ERRγ signaling in DAergic neurons; in fact, studies show that ERRγ activity can be regulated by phosphorylation and sumoylation events^88^. Synuclein could affect select ERRγ-dependent genes by influencing the post-translational state and, thus, transcriptional activity of ERRγ and/or its interacting proteins; it is interesting to note that stability and ubiquitination of ERRs can be regulated by Parkin^89^. ERRγ can also act as a sensor for reactive oxygen species^90^, the loss of which could be responsible for enhancing synuclein-mediated toxicity. Future experiments are needed to determine how synucleinopathy-induced cellular stress influences the transcriptional activity and/or post-translational modifications of components of ERRγ transcriptional complexes.

Surprisingly, we found that while complete deletion of *Esrrg* caused behavioral hypoactivity and cell loss with age, partial deletion caused ambulatory hyperactivity at baseline, with hyperactivity maintained through 21 months of age (*n* = 4/group; data not shown). Interestingly, we found an increase in striatal DA content in tamoxifen-treated i*Slc6a3Cre*;*Esrrg*^*fl/fl*^ mice at 12 months of age (Supplementary Fig. 10a), with no effects of *Esrrg* overexpression on striatal DA content (Supplementary Fig. 10b). Despite the difference in the models, mice with complete deletion did show hyperactivity prior to developing motor impairment (Fig. 2e). Of note, adolescent mice injected with the toxin 6-OHDA exhibit a similar ambulatory hyperactivity prior to neuronal death^91,92^, and LRRK2-overexpressing mice^93^, Parkin-deficient mice^94^, and A30P alpha-synuclein-overexpressing mice^95^ all exhibit increases in DA or ambulatory hyperactivity with no observed cell loss. Increased DA availability has been proposed as a possible contributor to synaptic toxicity^96^ and could be responsible for enhanced synaptic loss with *Esrrg* deficiency with PFF treatment. Based on these observations, we propose that partial deletion of *Esrrg* recapitulates an early stage of DAergic neuron impairment observed in PD, with increased DAergic tone and vulnerability to synaptic damage.

Considering all the information above, we propose that *Esrrg* is a critical regulator of mitochondrial, synaptic, and proteostatic processes in adult DAergic neurons. The findings of this study present and highlight the importance of synaptic and mitochondrial function in DAergic neurons as well as the relevance of *Esrrg* in regulation of DAergic neuron properties. Due to the existence of agonists of ERRγ, this knowledge can be used to develop therapeutic strategies for neurological disorders, especially ones that are associated with abnormal DAergic neurotransmission and cell loss.

## Methods

### Animal models

All experimental procedures were approved by the Institutional Animal Care and Use Committee of the University of Alabama at Birmingham and of Southern Research and performed in accordance with the Association for Assessment and Accreditation of Laboratory Animal Care. *Esrrg*^*fl/fl*^ mice were generously provided by A.K. (Johns Hopkins University) and J.A. (Swiss Federal Institute of Technology Lausanne). Breeding schemes were established using mice heterozygous for the *Esrrg* floxed allele to generate littermate controls for all mouse lines. For adulthood cell-specific DAergic deletion studies, mice with expression of tamoxifen-responsive Cre-recombinase driven by the *Slc6a3* promoter (Jackson Laboratories #016583) were used with nuclear translocation of Cre induced by 5-days of consecutive administration of tamoxifen per the Jackson Laboratory protocol (https://www.jax.org/research-and-faculty/resources/cre-repository/tamoxifen). For specific deletion of *Esrrg* from forebrain excitatory neurons, mice expressing Cre-recombinase under control of the empty spiracles homeobox1 (EMX-1) promoter (*Emx-1*;Cre Jackson Laboratories #005628)^97^ were crossed with *Esrrg* floxed mouse line. Cre-recombinase-expressing females were used for breeding, and all control mice were Cre-positive with wildtype *Esrrg* alleles. For ribosomal affinity purification RNA sequencing (BAC-TRAP) studies, mice expressing Cre-dependent (EGFP)-L10a fusion protein (L10a; Jackson Laboratories #022367) were maintained and crossed with mice heterozygous for *Esrrg* to generate *Esrrg*^*+/+*^;L10^+^ and *Esrrg*^*fl/fl*^;L10^+^ mice for injection with AAV-*ThCre* recombinase to elicit recombination in DAergic neurons (ref. ^98^). All experiments were conducted using male and female mice with initial injections at 3-months of age with the following timepoints post-injection as follows: (1) AAV:*Th*Cre injected mice: 1–6 months post-injection; (2) i*Slc6a3; Esrrg:* 1–9 months post-injection; (3) all PFF experimental mice: 1, 3 and 6 months post-injection; (4) *Esrrg* overexpression studies: 1, 3, and 6 months post-injection; (5) BAC-TRAP mice: 1 month post-injection. Experimenter was blind to genotype. All mice were maintained on a C57BL/6J genetic background and housed 2–5 in a cage at 26 ± 2 °C room temperature with food and water ad libitum.

### Small molecule fluorescence in situ hybridization (sm-FISH)

In mice, sm-FISH was performed using the Basescope fluorescent assay followed by the RNAscope Multiplex Fluorescent Assay (Advanced Cell Diagnostics/ACD, Newark, CA, USA)^15,99^. Mice were anesthetized with isoflurane and decapitated, and brains were removed, and flash frozen on powdered dry ice. In total, 20 μm sections were sectioned on a cryostat, collected onto SuperFrost Plus slides (Thermo Fisher Scientific), and immediately refrozen. For the Basescope assay, tissue was fixed with 4% paraformaldehyde followed by dehydration with ethanol and treated with hydrogen peroxide and protease III (ACD, Newark, CA, USA). Next, slides were incubated with Basescope probes for 2 h at 40 °C followed by amplification and incubation with BaseScope Fast RED A and B (ACD, Newark, CA, USA). Due to the small length of transcript used to detect recombination, BaseScope probes were custom designed to recognize exons 1–3 (217–264 bp) of *Esrrg* (NM_011935.3; ACD, Newark, CA, USA; list of all probes used in Supplementary Table 3). Subsequently, RNAscope was performed by incubating tissue in a mixture of probes for 2 h at 40 °C followed by fluorescent amplification. Slides were coverslipped with Prolong gold antifade mounting media containing DAPI (Thermo Fisher Scientific) and images were captured with a Nikon A1 + confocal microscope. All settings, including laser intensity, gain, offset, and zoom, were held constant across all groups for a given experiment. For RNAscope/Basescope experiments *n* = 4–8 animals/genotype and *n* = 2 sections per animal were imaged.

To assess the relative area of the cell occupied by sm-FISH fluorescent signal, image thresholding for individual channels and quantification of mean pixel density for a given area was performed using ImageJ^100–102^. Briefly, thresholds were set for individual channels for each experiment based on a wild-type control signal, and single-cell regions of interest were defined and circled using a cell marker (e.g., *Th* mRNA) to generate mean pixel density values for each gene using ImageJ. This method considers both the area of the neuron and the number of positive pixels for each gene to allow for semi-quantitative analysis of a gene of interest. For generation of representative images, tiff files were imported into Adobe Photoshop CS3 (Adobe, San Jose, CA), and adjustments to contrast, sharpness and brightness were held constant across experimental groups.

For a subset of experiments, immunofluorescence for phosphorylated α-synuclein (p-syn; Biolegend; antibodies are listed in Supplementary Table 4) was performed using a Mouse-on-Mouse kit (M.O.M. Vector laboratories) followed by the ABC kit (Avidin/Biotin Systems reagents per manufacturer’s instructions) to visualize p-syn-positive inclusions. Sections were incubated in p-syn antibody for 1 h at room temperature followed by a 30 min incubation in secondary conjugated to streptavidin (AMCA conjugate). Tissue was coverslipped with prolong gold antifade mounting media without DAPI (Thermo Fisher Scientific), and images were captured with a Nikon A1 + confocal microscope. In some cases, pseudo-coloring from a non-green channel into a green channel was used to enable visualization of the signal of interest (with no use of any type of nonlinear adjustment). Area of the cell occupied by signal for p-syn was estimated utilizing the same process as for sm-FISH quantification (above).

Human postmortem brain tissue was obtained from the Alabama Brain Collection, see Supplementary Table 5 for details. Tissue was fresh frozen and stored at −80 °C. The substantia nigra (SN) was sectioned fresh frozen at 20 µm on SuperFrost/Plus glass slides (cat# 1255015) (Fisher Scientific, Pittsburgh, PA) and placed at −80 °C. Post-mortem characteristics are listed in the table below. In situ hybridization was done following RNAscope Multiplex Fluorescent Regent Kit v2 (cat# 323110) per the manufacturer’s instructions (Advanced Cell Diagnosis, ACD). Tissue was treated for 2 h at 40 °C with the following combination of ACD probes: Hs-TH-C2 (cat# 441651), Hs-ESRRG-C3 (cat# 523271). After amplification steps, each channel was fluorescently labeled individually as follows: C2 probe - TSA Cyanine 3 Plus Evaluation Kit (cat# NEL744E001KT); C3 probe—TSA Cyanine 5 Plus Evaluation Kit (cat# NEL745E001KT) (PerkinElmer, Inc). Then, coverslips were added with Prolong gold antifade mounting medium containing DAPI (cat# P36962) (Thermo Fisher Scientific).

### Stereotaxic injections

Before surgery, a subcutaneous injection of buprenorphine (0.1 mg/kg; analgesic) and carprofen (3 mg/kg; anti-inflammatory) was administered. The mice were anesthetized with isoflurane (4–5% induction, 1–2% maintenance), immobilized in a stereotaxic apparatus (Stoelting), and kept on a heating pad through the surgery and recovery. Bregma was identified, and a small burr hole was made in the skull using a drill. Viruses included AAV9.rTH.PI.Cre.SV40 (AAV9:*ThCre*; AddGene# 107788), AAV5-h*Syn*-GFP-Cre (University of North Carolina Vector Core), AAV5:*Gfp*, or AAV5:*Esrrg* (Plasmids generated and cloned into AAV2 backbone with CAG promoter Addgene# 28014^103^ by Genscript; packaged into an AAV5 by Vectorbiolabs; AAV5:CAG-*Esrrg-*NM_011935.3-IRES-EGFP-WPRE; virus titer 2.0 × 10^12^ GC/ml). In total, 2 µl virus was bilaterally injected at a constant rate of 0.5 μl/min into the midbrain of *Esrrg*^*+/+*^ or *Esrrg*^*fl/fl*^ mice using the coordinates A/P: −3.0 M/L: ±1.5 D/V: −4.6. The syringe was withdrawn following a four-minute pause. For AAV-mediated deletion of *Esrrg*, AAV9:*ThCre* was injected into the midbrain of *Esrrg*^*fl/fl*^ mice to induce recombination in cells expressing TH. Overexpression experiments were conducted using an AAV5:CAG-*Gfp* or AAV5:CAG-*Esrrg-*IRES-EGFP-WPRE. For intrastriatal injection of preformed α-synuclein fibrils PFF (see below); fibrils were thawed and sonicated using a probe tip sonicator (Fisher, FB120110) for 30 s total time, with 1 s pulses at 30% amplitude. At 3 months of age, 2 days following tamoxifen treatment, i*Slc6a3Cre*;*Esrrg*^*+/+*^ or i*Slc6a3Cre*;*Esrrg*^*fl/fl*^ mice were injected with PFF or monomer. For overexpression studies, wild-type C57BL/6 mice received striatal injections with either PFF or monomer and nigral injected with either AAV5:*Esrrg* or AAV5:CAG-*Gfp* control at 3 months of age. Animals (*n* = 6–8/group; harvested 1, 3 or 6 months post-injection) were bilaterally injected into the dorsolateral striatum (A/P: +0.2 M/L: +2.0 D/V: −2.6) with 2 μl per side of 300 μM sonicated fibrils or 300 μM monomeric alpha-synuclein at a constant rate of 0.5 μl/min. The syringe was left in place for 4 min followed by a slow withdraw.

### Behavior

All behavior was conducted during the lights-on period with and in the presence of a white noise machine to reduce any startling responses to outside factors. In some cases, mice were tested in open field and pole assays at multiple time points. Before all tests, mice were placed in the behavioral testing room to acclimate for 30 min.

### Open field

For assessment of baseline activity (ref. ^16^), animals were placed in a square apparatus (27.31 cm Length × 27.31 cm width × 20.32 cm height) consisting of 48 infrared beams (Med Associates) for 30 min. For behavioral assessment with stimulation of DA release, mice were injected intraperitoneally (IP) with 5 mg/kg of *d*-amphetamine and allowed to freely explore the open field box for a total of 60 min. This approach was used to reveal subtle differences in DAergic neurons, as used previously in PINK1 and Parkin knockout models^53–55^. Data were collected with Open Field Activity Software (Med Associates) in 1 min intervals over the test period. For experiments to rescue baseline hypoactivity in the 14 month post-AAV:*ThCre*;*Esrrg*^*flfl*^-injected mice, l-3,4-dihydroxyphenylalanine (L-DOPA; Millipore Sigma D1507) was injected IP at a 6 mg/kg concentration with Benserazide (12 mg/kg; Millipore Sigma B7283), and mice were allowed to explore the open field box for 60 min.

### Pole assay

Mice were placed on the top of a vertical pole (60 cm vertical pole tall, diameter of 1 cm, mounted on a triangular base stand, tightly wrapped with chicken wire) with the bottom placed in the home cage. Recording started when the animal was placed on top of the pole. The time to turn completely downward and total time to descend to the cage floor were recorded. If the animal paused while descending, the trial was repeated. If the animal fell off the pole, the maximum time of two minutes was assigned. An average of 3 trials per animal was recorded per mouse. The pole was cleaned with ethanol between each mouse. For experiments to rescue the pole assay deficit in 14 month post-AAV:*ThCre*;*Esrrg*^*flfl*^-injected mice, L-DOPA (Millipore Sigma D1507) was IP injected at a 6 mg/kg concentration along with Benserazide (Millipore Sigma B7283) at 12 mg/kg and mice were allowed to descend the pole as described above, following testing in open field.

### Immunofluorescence

All antibodies are listed in Supplementary Table 4. It is important to note here that for all fresh frozen sections, no EGFP was visible, so secondary antibodies and sm-FISH probes could be used in the FITC channel. For striatal section analysis, slides frozen and sectioned for sm-FISH were fixed in 4% PFA and washed in PBS before blocking for 1 h with 10% serum from the host of the secondary antibody in PBS. Slides were then incubated with the primary antibody in 3% BSA and 0.3% Triton X-100 (Sigma- Aldrich) in PBS at room temperature for 2 h. The following primary antibodies were used: anti-DA active transporter (1:200 concentration; DAT; Millipore Sigma), and anti-TH (1:200 concentration; TH; Millipore Sigma). Slides were then rinsed and incubated with the corresponding fluorescence-conjugated secondary antibodies (1:1000 concentration; Jackson ImmunoResearch) for 1 h at room temperature in 5% serum, 3% BSA, and 0.3% Triton X-100 in PBS. Sections were coverslipped using Prolong Antifade Gold without DAPI (Thermo Fisher Scientific) and stored at 4 °C. Images were captured using the BIORAD ChemiDoc MP imaging system and analyzed using ImageJ to determine relative intensity in the dorsolateral striatum and olfactory tubercle, with subtraction of background signal (cortex; no group differences were observed in cortical fluorescence intensity, Supplementary Fig. 2). To generate representative images, tiff files were imported into Adobe Photoshop CS3 (Adobe, San Jose, CA) and adjustments to contrast, sharpness and brightness were held constant across experimental groups.

For immunofluorescence for DAergic neuron cell counts (see sampling rate, below), slides frozen for sm-FISH were fixed in 4% PFA and washed in PBS before blocking for 1 h with 10% serum from the host of the secondary antibody in PBS. Slides were then incubated with the primary antibodies (Anti-TH, 1:200 concentration; anti-neuronal nuclear antigen, 1:200 concentration, Millipore) in 3% BSA and 0.3% Triton X-100 (Sigma- Aldrich) in PBS at room temperature for 2 h. Slides were rinsed and incubated with the corresponding fluorescence-conjugated secondary antibodies (1:1000 concentration; Jackson ImmunoResearch) for 1 h at room temperature in 5% serum, 3% BSA, and 0.3% Triton X-100 in PBS. For slides from mice injected with PFFs, Mouse-on-Mouse kit (M.O.M. Vector laboratories) followed by the ABC kit (Avidin/Biotin Systems reagents per manufacturer’s instructions) to visualize p-syn-positive inclusions. Sections were incubated in p-syn antibody for 1 h at room temperature followed by a 30 min incubation in secondary conjugated to streptavidin (AMCA conjugate). Slides were then washed in PBST 3 × 2 min and then incubated in NEUROTRACE 640/660 Deep Red Fluorescent Nissl Stain 1:2000 concentration for 20 min at RT. Sections were then washed and coverslipped using Prolong Antifade Gold without DAPI (Thermo Fisher Scientific) and stored at 4 °C until imaged by the Nikon A1 + confocal microscope. To generate representative images, tiff files were imported into Adobe Photoshop CS3 (Adobe, San Jose, CA) and adjustments to contrast, sharpness and brightness were held constant across experimental groups.

### DAergic neuron cell count estimation

To estimate the relative number of TH+ neurons in the SN across groups, one section at 8 levels per mouse was stained with TH, NeuN, and p-syn antibodies, followed by a fluorescent Nissl stain (Supplementary Fig. 12). Images were then captured on Nikon A1 + confocal microscope and only neurons that were positive for TH, NeuN and Nissl were counted. In mice injected with PFFs, neurons that were positive for p-syn were also counted to allow for calculation of inclusion load. In mice injected with AAV:*Th*Cre; only 1 hemisphere was counted because the other hemisphere was used for electron microscopy analysis. Neuron counts are reported as raw values because standard stereological calculations could not be performed with sections at 20 µm.

### Immuno-electron microscopy

All antibodies are listed in Supplementary Table 4. Fresh brains were immersed in cold 4% paraformaldehyde and 1% glutaraldehyde in 0.1 M phosphate buffer (PB), pH 7.4. The tissue was stored in this fixative at 4 °C until it was further subdissected. The SN was dissected out of the brains and sectioned on a vibratome at a thickness of 40 µm in series of six. The tissue was stored in 0.1 M PB at 4 °C until it was processed; three or four sections per animal were selected from a random series for the immunohistochemical localization of TH. Immunohistochemistry (IHC) was performed with free-floating sections from 6 mice/genotype. First tissue sections were rinsed 5 × 5 min in phosphate buffered saline (1X PBS), a solution of 1% sodium borohydride in 0.01 M PBS was added to the sections for 15 min at room temperature (RT) while agitating. Next, sections were incubated in a solution of 5% hydrogen peroxide in 0.01 M PBS for 30 min at RT while agitating then washed 4 × 5 min in 0.01 M PBS. Sections were then incubated in a solution of 10% normal donkey serum in 0.01 M PBS for 1 h at RT while being agitated. Sections were incubated on an orbital shaker in 3% normal donkey serum in 0.01 M PBS with Anti-TH primary antibody (1:200, AB152) overnight at 4 °C. Then, sections were washed in 0.01 M PBS 4 × 5 min followed by incubation in 3% normal donkey serum in 0.01 M PBS with HRP-conjugated donkey anti-rabbit secondary antibody (1:500, Jackson Immuno Research 211-032-171) for 45 min RT. Sections were washed in 0.01 M PBS 4 × 5 min. To visualize staining for electron microscopy (EM), sections were incubated for 2 min in a 3, 3′-diaminobenzidine solution (2 drops of Reagent 1, 4 drops of Reagent 2, 2 drops of Reagent 3, and 2 drops of the Nickel Solution were added to 5 ml of sterile water, Vector Laboratories, SK-4100).

After desired staining was achieved, sections were washed 4 × 5 min in 0.01 M PBS and prepared for EM using standard techniques^104^. The sections were then immersed in 1% osmium tetroxide in PB at room temperature in the dark for 1 h, rinsed 4 × 5 min each in PB, then dehydrated at room temperature in increasing concentrations of EtOH. After that, the tissue was stained en bloc in a 1% uranyl acetate solution in 70% EtOH for 1 h followed by dehydration in increasing concentrations of EtOH, 100% propylene oxide, epon resins, and heated at 60 °C for 72 h.

Photomicrographs of each section were taken at ×10 on a light microscope; these sections served as a reference for selecting areas to block out for EM analysis. After choosing a region in the SN, blocks were glued to epon beem capsules and semi-thin sectioned at 100 µm. Thin sections at a thickness of 90 nm were then collected on slot grids and viewed with a Hitachi electron microscope. Immunolabeled neurons from one or two different areas of the SN were photographed on the electron microscope at a magnification of ×4000. Then cytoplasm from each neuron was photographed at a magnification of 15,000. Low power electron micrographs (×4000) were used to determine the area of the cytoplasm for each neuron. All immunoreactive neurons were photographed from a region with the goal of sampling at least 20 neurons; if necessary, another region was blocked and photographed to reach that goal. Mitochondria were counted and the cross-sectional diameter measured in ×15,000 micrographs. Measurements were taken using Adobe Photoshop. Three measurements were determined: the number of mitochondria per neuron, the number of mitochondria per area of cytoplasm per neuron and the diameter of each mitochondrion. An average of 21.33 neurons and 624.42 mitochondria were counted per mouse. The mouse groups were coded to keep the investigators blind to genotype.

### Generation of preformed alpha-synuclein fibrils

Monomeric mouse alpha-synuclein was purified (ref. ^105^) followed by the generation of preformed fibrils by incubating monomeric alpha-synuclein (350 μM) in PBS (137 mM NaCl, 2.7 mM KCl, 8 mM Na2HPO4 2 mM KH¬2PO4) at 37 °C with constant agitation of 1000 rpm for 7 days^106^. The formation of amyloid fibrils was confirmed by the Thioflavin T fluorescence assay and imaging with a Tecnai G2 Spirit TWIN transmission electron microscope (FEI Company, Hillsboro, OR). Fibrils were aliquoted and stored at −80 °C until use. Once fibrils were thawed for use, they were stored at room temperature and used within 1 week.

### Quantitative reverse transcriptase PCR

Mice were anesthetized with isoflurane prior to decapitation. Brains were rapidly removed, and microdissected brain regions were flash frozen on dry ice and stored at −80 °C until use. For transcriptional analysis of genes (Supplementary Table 6), midbrain was homogenized in TRIzol using a Bead Ruptor 12 from (Omni International) and RNA was isolated using the TRIzol/choloform-isopropanol method following the manufacturer’s instructions (Invitrogen). RNA concentration and purity were determined using a NanoDrop 2000 (Thermo Fisher Scientific). Equivalent amounts of RNA (1 μg) were treated with DNase I (Promega) at 37 °C for 30 min and DNase Stop solution at 65 °C for 15 min. RNA was reverse-transcribed using the High-Capacity cDNA Archive Kit (Thermo Fisher Scientific). Transcripts were measured using mouse-specific primer/probe sets (Supplementary Table 6) from Applied Biosystems and JumpStart Taq Readymix (Sigma-Aldrich) using a protocol with an initial ramp step (2 min, 50 °C; 10 min, 95 °C) and 40 subsequent cycles (15 s, 95 °C; 1 min, 60 °C). Relative concentration of transcript was calculated compared with a standard curve generated from pooled cDNA samples (1, 1:5, 1:10, 1:20, 1:40; calibrator method). These values were normalized to β-actin and expressed as ratio to control samples ± standard error of the mean (SEM). For graphs in the main manuscript body that do not show individual datapoints, datapoints are included in Supplementary Fig. 11.

### BAC-TRAP

*Esrrg*^*+/+*^*;*L10+ and *Esrrg*^*fl/fl*^;L10+ mice were injected with 2 ul of AAV.rTH.PI.Cre.SV40 (AddGene) into the midbrain to cause recombination for a total of 8 animals (16 nigra) per sample. *Esrrg*^*+/+*^*;*L10+ mice with no AAV-cre injection were used as negative controls. Mice were sacrificed 1 month post-injection, and midbrain was dissected and flash frozen in dry ice. RNA immunoprecipitation and isolation was performed (ref. ^98^). Pooled midbrain were homogenized and lysed to allow for immunoprecipitation of gfp-positive DAergic neurons to anti-eGFP (Cat# Htz-GFP-19F7,RRID:AB_2716736/Cat# Htz-GFP-19C8, RRID:AB_2716737,Memorial Sloan-Kettering Monoclonal Antibody Facility) bound Protein G beads (Dynabeads MyOne Streptavidin T1 Thermo Fisher Scientific Cat# 65602). Beads were then washed and collected on a magnet, and the RNA from the actively translating ribosomes was isolated for RNA (Absolutely Total RNA Purification Kits, Agilent Cat# 400753). cDNA was generated from the resulting RNA to confirm both enrichment of *Th* DAergic population and the reduction in *Esrrg* transcript. Once confirmed, samples were shipped to Novogene for sequencing. Sequencing was conducted using an Illumina Hiseq4000 obtaining 150PE reads and using a PolyA selected, non-strand specific library to generate 20 M raw reads.

### RNA-seq analysis

Raw sequence reads were first trimmed with Trim Galore (version 0.6.6) to remove primer adapter contamination. STAR (version 2.7.7a) was used to align the trimmed RNA-Seq fastq reads to the mouse reference genome (GRCm38 p6, Release M24) from Gencode^107^. Following alignment, HTSeq-count (version 0.11.2) was used to count the number of reads mapping to each gene^108^. Normalization and differential expression were then applied to the count files using DESeq2^109^ (version 1.30.1), using ±1.5 fold change and adjusted *p* value of 0.05 as a cutoff, with comparison of WT2-4 to FL2 and FL4 (awaiting GEO accession number). DEXSeq (version 1.36.0) was also used to identify differential exon usage in the exon count data from these samples^110^. Gene lists are included in Supplementary File 1.

### Flag-*Esrrg* overexpression in SH-SY5Y’s

Overexpression of *Esrrg* in SH-SY5Y’s was achieved through infection with recombinant Ad CMV 3xFlag-*Esrrg*. To create this adenovirus, first a construct containing the coding sequence for *Esrrg* (*Esrrg*-NM_011935.3) with a 3x-flag-*Esrrg* fragment was cloned into Invitrogen Gateway pENTR 2B dual selection vector (A10463) and resulting plasmid was used for LR recombination with pAd CMV/V5-DEST (Invitrogen, V49320). Final pAd CMV 3xFlag *Esrrg* plasmid linearized by PacI was used to transfect 293 cells to obtain Ad CMV 3xFl-*Esrrg* virus. Viral preps were produced and purified by double Cs banding in the UAB Vector and Virus Core. The titer of purified viral prep was determined by OD260 and by plaque assay on 293 cells. SH-SY5Y cells were infected with purified Ad CMV 3xFlag-*Esrrg* to induce overexpression of *Esrrg* and control cells were infected with Ad CMV GFP^29^. Cells from both groups were collected 24 h later for RNA sequencing experiments. Gene lists are included in Supplementary File 1.

### Generation of cell-type-specific protein-protein-interaction networks

Data imported from the BIOGRID database were filtered to include only interactions for human proteins from 2 or more publications or experimental methods, yielding a filtered graph of 4408 nodes and 10,061 edges, with a node representing a distinct genetically encoded protein and an edge representing an interaction between two proteins^111^. Before performing the analyses, any gene which was not present in the DAergic neuron cell type as defined by Dropvoz.org^30^ (SN_4.1–4.9) was removed from the Biogrid interactor list to favor cell-type specificity of the PPI network. These data were then used to instantiate an undirected graph using the NetworkX python library^112^.

We identified the communities for each of the input genes which is defined as the subgraph induced by the set of nodes 2 or less edges away from a gene. Next, we calculated the membership number (membership number is equal to the number of communities containing each gene) for each identified gene. These numbers were then normalized to a proportion by dividing the membership number by the number of communities. To generate expected membership proportions and significance values to filter out genes overrepresented in the source data, we performed Monte Carlo simulations to calculate community membership proportions for 1000 random sets of genes of size equal to the input gene list, providing a mean membership proportion with standard deviation for each network node. Fold enrichments (connectivity values) were calculated by dividing the actual membership proportion by the expected membership proportion for each gene using methods adapted from the Panther overrepresentation tests^113^. Lists were filtered to exclude any genes with membership proportion less than 3 standard deviations above the mean expected membership proportion, and to exclude any genes with a membership number of 1 (e.g., only existing in one community, which may have still been significantly overrepresented). The list of genes was then expanded by adding any missing genes from the input list (nodes may have been dropped due to no interactions fitting the initial graph criteria).

To prioritize PPI-identified targets, we generated a composite score for each gene based on three variables: (1) connectivity score in PPI network (“Connectivity”; NeuroInitiative, Jacksonville, FL), (2) relative enrichment within DAergic neurons compared to other cell types (“Relative Enrichment in Cell Type”; determined using Saunders et al.^30^), and (3) raw abundance in DAergic neurons (“Expression”; as reported in Saunders et al.^30^). Each variable value was normalized to the highest value within that category across the 504 PPI genes, making the highest possible value for each variable 1.0. A composite score was assigned to each gene based on the sum of their normalized variable values, with a maximum composite score of 3.0. Based on this composite score, we selected the top 25 targets to graphically display across the three criteria (Fig. 7h). (Connectivity Value/Highest Connectivity Value) + (Expression Value/Highest Expression Value) + (Relative Enrichment Value/Highest Enrichment Value) = Maximum Composite Score of 3.0. Gene lists are included in Supplementary File 1.

### Gene ontology (GO) analysis

Convergent molecular functions were explored with Enrichr^114–116^ using the GO molecular function tool.

### Curation of PD risk genes for transcriptional comparisons and PPI network generation

Lists of GWAS-implicated genes were generated from predicted genes in the vicinity of risk loci^117–119^, and QTL gene lists were compiled using identified risk single-nucleotide polymorphisms (SNPs) as seed SNPs for exploration of expression or splicing differences associated with each loci in gtexportal.org v8. Combined (GWAS + QTL) lists were filtered to only include genes with mouse homologs and expressed in mouse brain (Dropviz.org^30^). Gene lists are included in Supplementary File 1.

### Western blot

All antibodies are listed in Supplementary Table 4. Brains sectioned for sm-FISH were collected by scraping the dorsolateral striatum and cortex, pooling 8 sections per sample. Samples were placed in RIPA buffer (150 mM NaCl, 50 mM Tris, 1% Triton X-100, 0.1% SDS, 0.5% deoxycholic acid, pH 8.0), and homogenized using the Omni Bead Ruptor Homogenizer (OMNI International)^29^. Total protein concentration was determined with a bicinchoninic acid protein assay kit (Thermo Fisher Scientific), and absorbance was measured at 540 nm. Protein was then denatured in sample buffer (62.5 mM Tris-HCl, 20% glycerol, 2% SDS, 5% β-mercaptoethanol, 1 mg/ml bromophenol blue; pH 6.8) at 95 °C and equivalent amounts of protein were loaded into 4–20% Mini-PROTEAN® TGX™ precast gels (Bio-Rad cat# 4561093, Hercules, California, USA). Next, protein was transferred onto PVDF membranes which were then blocked for 1 h with 5% milk in Tris-buffered saline (TBS; pH 7.6) with 1% Tween (TBS-T). Following, membranes were probed with an antibody for TH (1:1000; EMD Millipore AB152) and actin (1:20,000 MAB1501, EMD Millipore) in 5% IgG-free bovine serum albumin (BSA; Jackson ImmunoResearch) in TBS-T overnight at 4 °C. Following incubation in HRP-conjugated anti-mouse and anti-rabbit secondary antibodies (Jackson ImmunoResearch cat# 115-035-174 and 211-032-171) in 5% milk in TBS-T for 1 h at room temperature, membranes were placed in Clarity™ Western ECL HRP substrate (Bio-Rad) for 5 min and imaged using the ChemiDoc MP Imaging System (Bio-Rad). The optical density of bands was calculated after background subtraction using Image Studio Lite (LI-COR, Lincoln, NE, USA). All bands were first normalized an internal control which was the same across all gels and then normalized to actin and expressed as mean optical density. All blots derive from the same experiment and were processed in parallel.

### High-performance liquid chromatography

Animals were sacrificed, striatum was microdissected and flash frozen in 2-methylbutane with dry ice, and samples were kept at −80 °C until processed by the Vanderbilt Neurochemistry Core^16^. Striata were homogenized in 100–750 ml of 0.1 M TCA, which contains 10^−2^ M sodium acetate, 10^−4^ M EDTA, 5 ng/ml isoproterenol (as internal standard) and 10.5% methanol (pH 3.8). The supernatant was separated by centrifugation, removed, and stored at 280 °C until use. Biogenic amines were identified by a specific HPLC assay utilizing an Antec DECADE II electrochemical detector (DataApex, Prague, Czech Republic) operated at 33 °C with an oxidation of 0.4. 20 ml samples of the supernatant were injected using a Waters 717+ Autosampler (Meadows Instrumentation Inc., Bristol, WI, USA) onto a Phenomenex Nucleosil C18 HPLC column (15064.60 mm; Torrance, CA, USA). Biogenic amines were eluted with a mobile phase consisting of 89.5% 0.1 M TCA, 10^−2^ M sodium acetate, 10^−4^ M EDTA and 10.5% methanol (pH 3.8). Solvent was delivered at 0.6 ml/min using a Waters 515 HPLC pump (Meadows Instrumentation). Using this HPLC solvent, biogenic amines elute in the following order: noradrenaline, 3-Methoxy-4-hydroxyphenylglycol (MHPG), adrenaline, 3,4-Dihydroxyphenylacetic acid (DOPAC), DA, 5-Hydroxyindoleacetic acid (5-HIAA/HVA), 5-hydroxytryptamine (5-HT), and 3-methoxytyramine (3-MT). HPLC control and data acquisition were managed by Empower software (Orlando, FL, USA).

### Statistics

All statistical analyses were performed using GraphPad Prism v9.0.2 (GraphPad Software, San Diego, CA, USA). Data are presented as the mean ± SEM. Individual data points are shown on graphs for cases with *n* = 3–10. All other graphs with individual data points can be found in Supplementary Fig. 11. All data with two independent variables or more (i.e., sm-FISH analysis, behavioral analysis, qPCR analysis, IF analysis) were analyzed using parametric two-way ANOVA followed by Tukey’s multiple comparisons if groups were equal or mixed-effects analysis ANOVA with Sidak’s multiple comparisons if groups were unequal following tests for normality using the Kolmogorov–Smirnov test and QQplots, with tests for variance across groups using the Bartlett’s test. If these assumptions were not met, data were then analyzed using a Kruskal–Wallis followed by Dunn’s multiple comparisons test. For analyses of two groups (i.e., transcript analysis, sm-FISH, Electron Microscopy, HPLC), data were analyzed using a two-tailed unpaired *t*-test. One-sided *t*-tests were used in cases in which previous reports and/or BAC-TRAP sequencing demonstrated directionality of response (rt-PCR, FISH). Significant differences were described in the graph when the *p* value was less than 0.05 and assumed at *p < 0.05, **p < 0.01, ***p < 0.001, and ****p < 0.0001. Outliers were identified using ROUT with *Q* set at 1%.

## Supplementary information

Supplementary Data Files

Dataset 1

## Data Availability

BAC-TRAP (GSE198216), *Esrrg*-flag overexpression (GSE198217), PGC-1α overexpression (GSE151499; 10.1016/j.neuroscience.2021.10.007), PFF study (10.1073/pnas.191390411), https://Dropviz.org (10.1016/j.cell.2018.07.028), PD GWAS “seed” SNPs (10.1038/ng.3955, 10.1016/S1474-4422(19)30320-5, 10.1038/ng.3043), QTL (any gene changed at the expression or splicing level in GTEx portal v.8). Unique reagents are available upon request.
