# Supplementary material for: Estrogen-related receptor gamma regulates mitochondrial and synaptic genes and modulates vulnerability to synucleinopathy
Source: NPJ Parkinsons Dis. 2022 Aug 18;8:106. doi: 10.1038/s41531-022-00369-w (PMC9388660; doi:10.1038/s41531-022-00369-w)
Supplement: Supplementary file 1 — Supplementary Data Files [file 41531_2022_369_MOESM1_ESM.pdf]

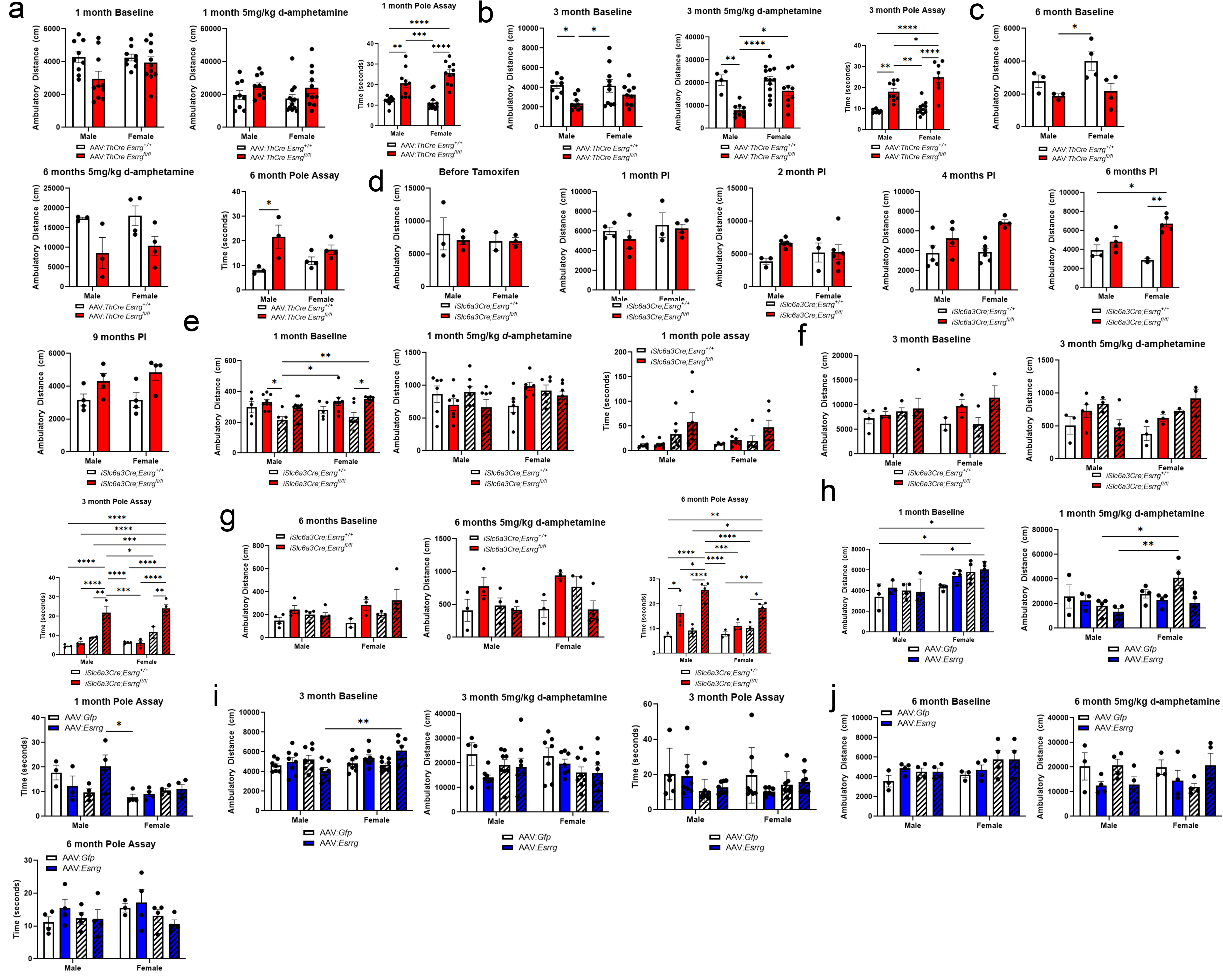

Supplementary Figure 1. All behavioral data split out by sex. Error bars represent ±SEM.

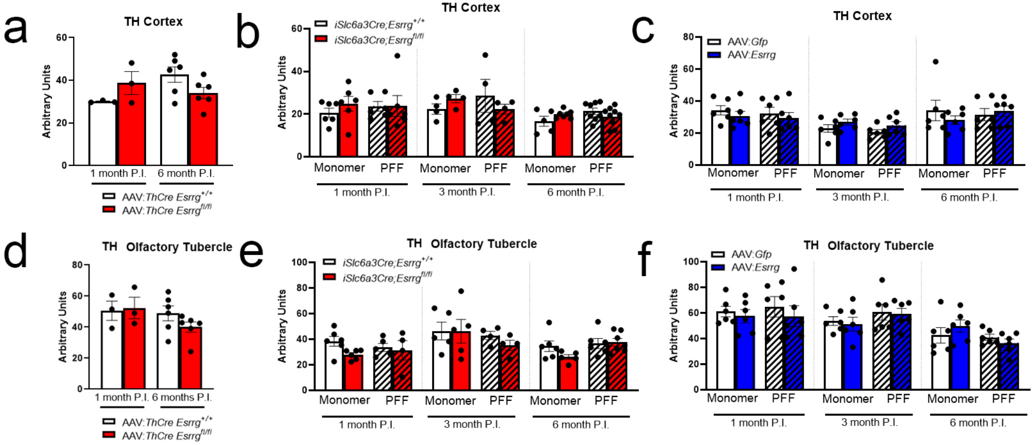

Supplementary Figure 2. Quantification of TH in the cortex or olfactory tubercle in *Esrrg*$^{+/+}$ and *Esrrg*$^{fl/fl}$ mice injected with AAV:*ThCre* into the midbrain (**a, d**), i*Slc6a3Cre;Esrrg*$^{+/+}$ and i*Slc6a3Cre;Esrrg*$^{fl/fl}$ mice 1, 3, and 6 months post-injection (P.I.) with PFFs or monomer (**b, e**), or AAV-*Gfp* or AAV-*Esrrg* 1, 3, and 6 months P.I. with PFFs or monomer (**c, f**). (*n*=3-8/group; mixed-effects analysis with Sidak's *post-hoc* analysis at each time-point or one-way ANOVA with Tukey's *post-hoc* analysis). Error bars represent ±SEM.

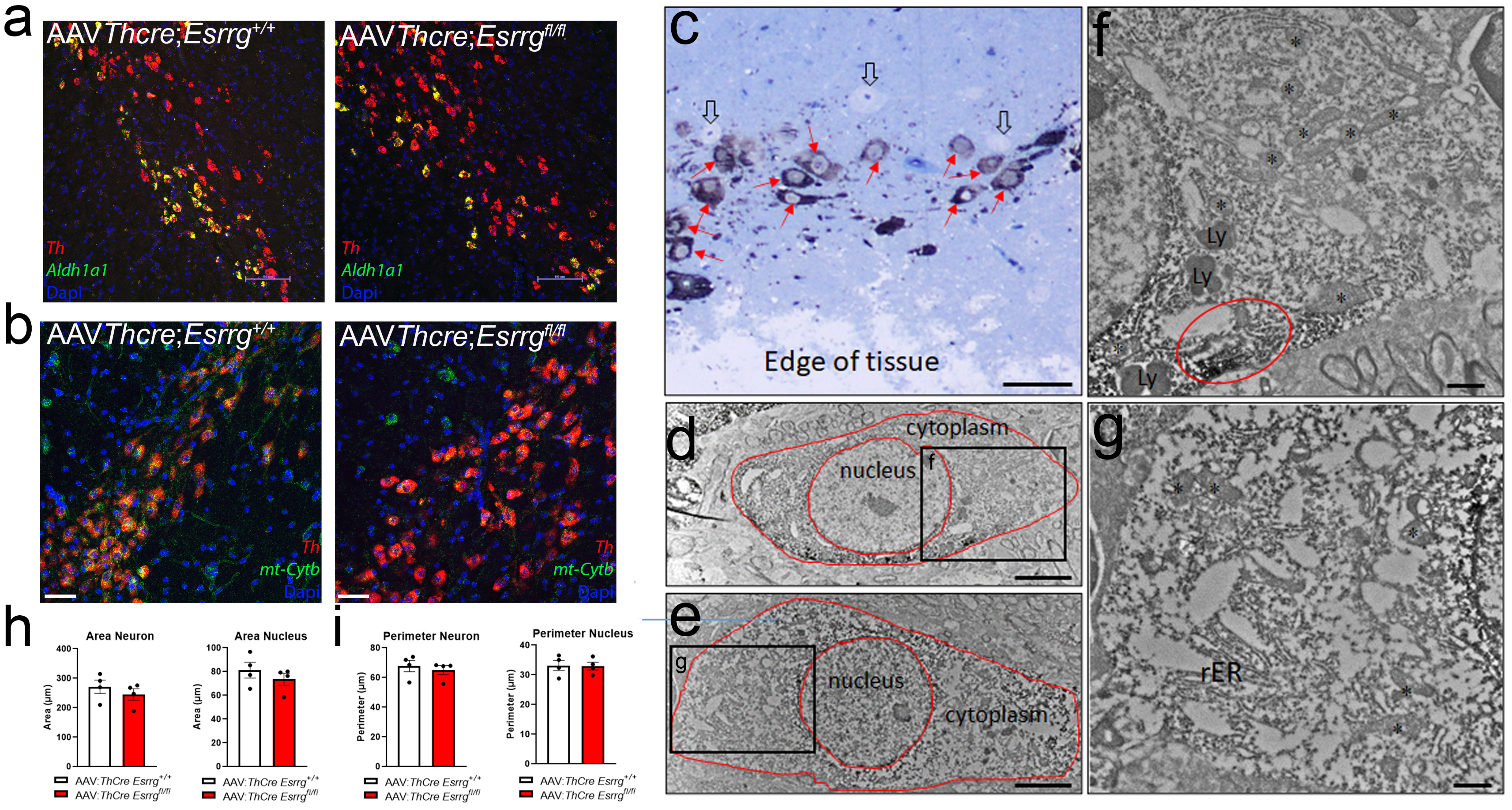

Supplementary Figure 3. a,b. Representative sm-FISH images for *Aldh1a1*, *Th*, and *mt-cytb* in DAergic neuron-specific knockout of *Esrrg* for graphs in Figure 2. **c.** Representative image from a semithin section used to identify immunolabeled neurons. Red arrows: neurons immunoreactive for tyrosine hydroxylase. Empty black arrows: local neurons without staining. **d-g.** Examples of electron micrographs used to generate data in figure 2. **d.** Example of an immunostained neuron with definition of the nucleus and cytoplasm in red. Boxed area corresponds to panel **f.** **e.** Example of an immunostained neuron with boxed area corresponding to rough endoplasmic reticulum, enlarged in panel **g.** **f.** boxed area from panel **d.** Ly:lysosome; *= mitochondrion; red circle: DAB immunoreactivity. **g.** Boxed area from panel **e.***= mitochondrion; rER=rough endoplasmic reticulum. **h,i.** Area or perimeter of the neuron and nucleus from all cells analyzed for EM. Scale bars correspond to **a)** 100µm **b)** 50µm **c)** 25µm; **d,e)** 5µm; **f,g)** 1µm. Error bars represent ±SEM.

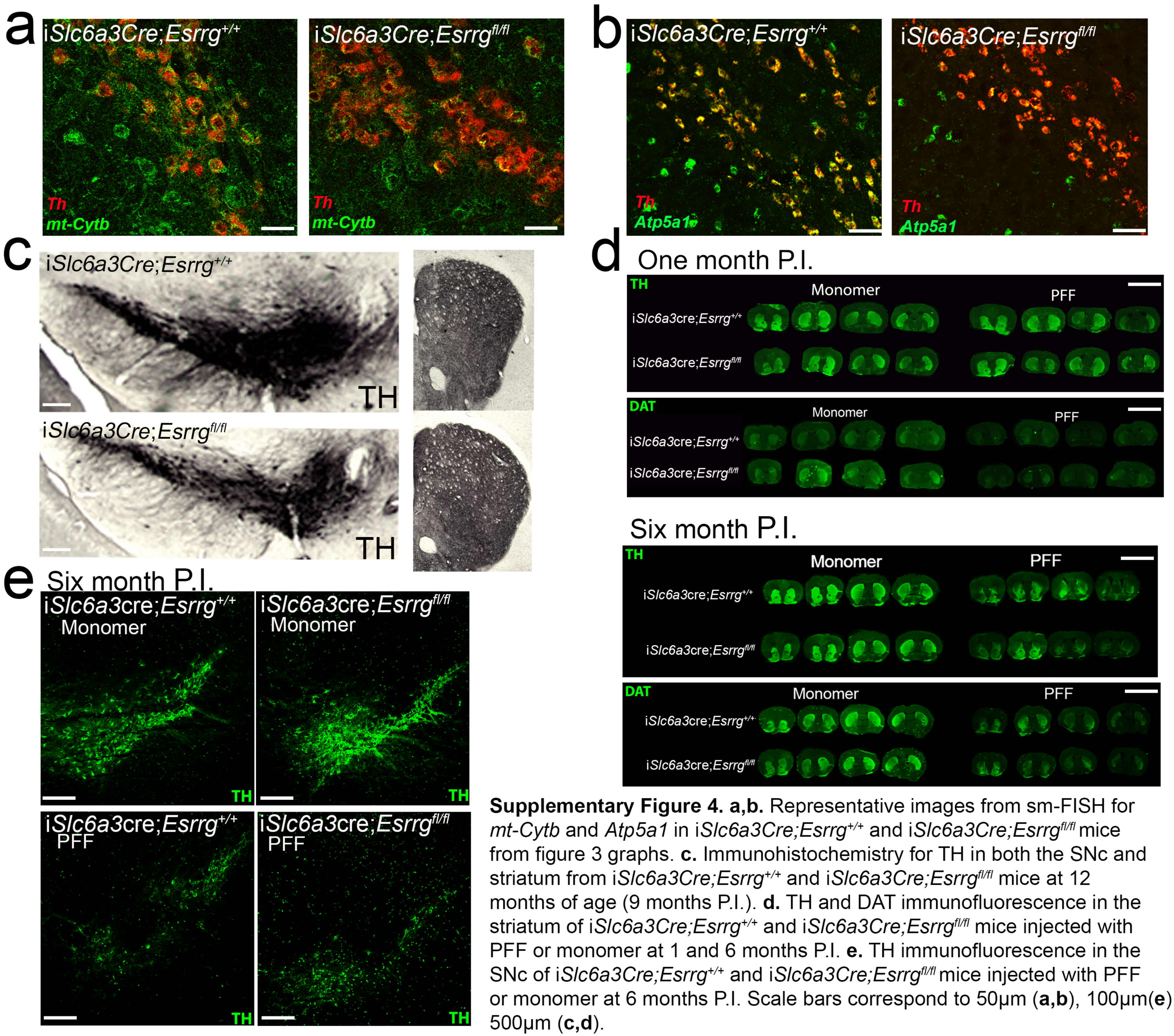

Supplementary Figure 4. a,b. Representative images from sm-FISH for *mt-Cytb* and *Atp5a1* in i*Slc6a3Cre;Esrrg*$^{+/+}$ and i*Slc6a3Cre;Esrrg*$^{fl/fl}$ mice from figure 3 graphs. **c.** Immunohistochemistry for TH in both the SNc and striatum from i*Slc6a3Cre;Esrrg*$^{+/+}$ and i*Slc6a3Cre;Esrrg*$^{fl/fl}$ mice at 12 months of age (9 months P.I.). **d.** TH and DAT immunofluorescence in the striatum of i*Slc6a3Cre;Esrrg*$^{+/+}$ and i*Slc6a3Cre;Esrrg*$^{fl/fl}$ mice injected with PFF or monomer at 1 and 6 months P.I. **e.** TH immunofluorescence in the SNc of i*Slc6a3Cre;Esrrg*$^{+/+}$ and i*Slc6a3Cre;Esrrg*$^{fl/fl}$ mice injected with PFF or monomer at 6 months P.I. Scale bars correspond to 50µm (**a,b**), 100µm(**e**), 500µm (**c,d**).

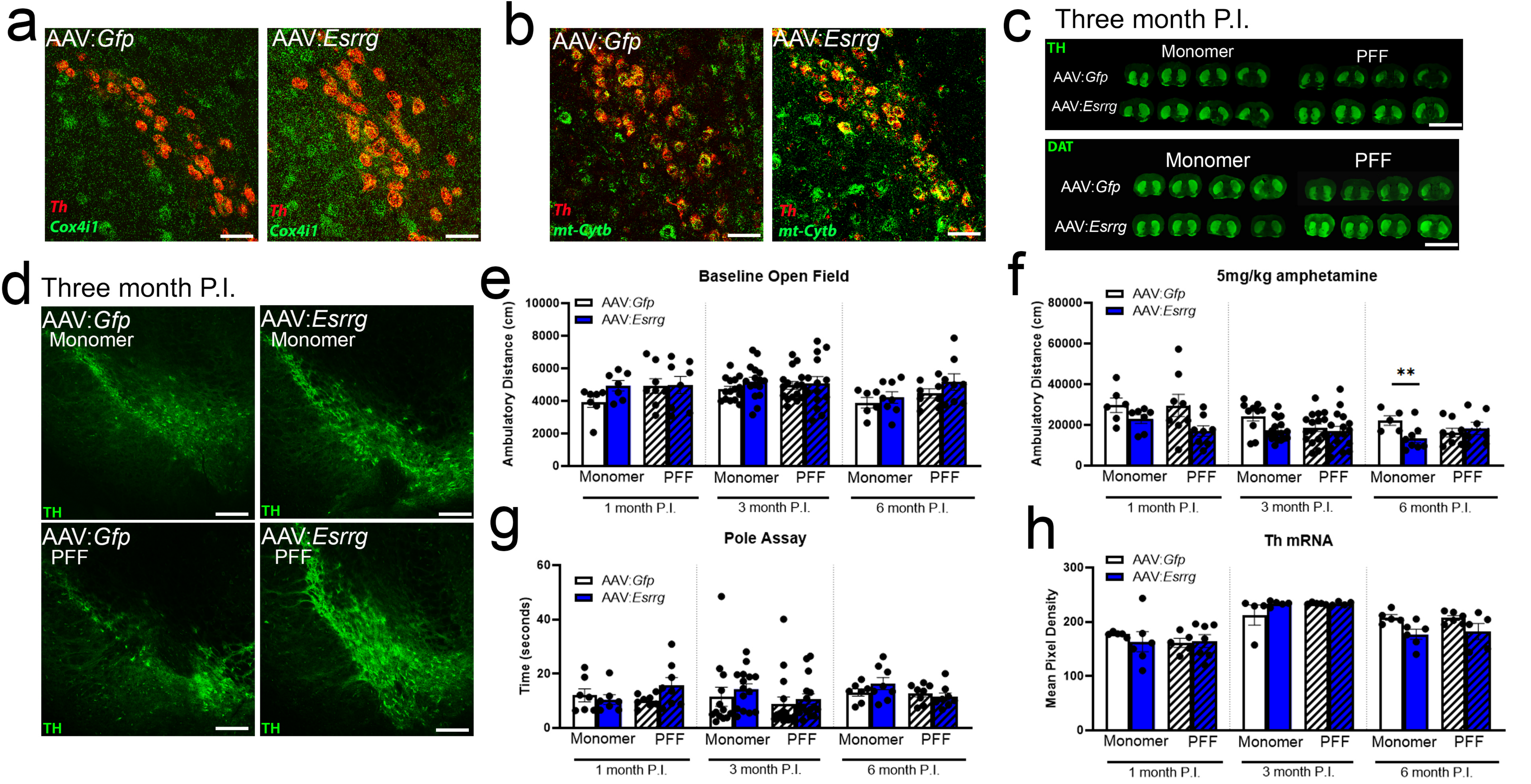

Supplementary Figure 5. a,b. Representative sm-FISH images for *Cox4i1* and *mt-cytb* from mice injected with AAV-*Gfp* or AAV-*Esrrg*. c. Representative images for TH and DAT immunofluorescence from the striatum at 3 months P.I. **d.** Representative images of TH immunofluorescence in the SNc of mice injected with AAV-*Gfp* or AAV-*Esrrg* and PFFs or monomer at 3 months P.I. **e-g.** Behavioral assessment using baseline open field, open field following an injection of *d*-amphetamine, and pole assay in mice injected with AAV-*Gfp* or AAV-*Esrrg* and PFFs or monomer at 1, 3, and 6 months P.I. (n=7-16/group; mixed-effects analysis with Sidak's post-hoc analysis at each time-point **p<0.01). **h.** sm-FISH for *Th* in mice injected with AAV-*Gfp* or AAV-*Esrrg* and PFFs or monomer at 1, 3, and 6 months P.I. (n=7-16/group; mixed-effects analysis with Sidak's post-hoc analysis at each time-point). Scale bars correspond to 50μm (**a**,**b**), 100μm (**d**), 500μm (**c**). Error bars represent ±SEM.

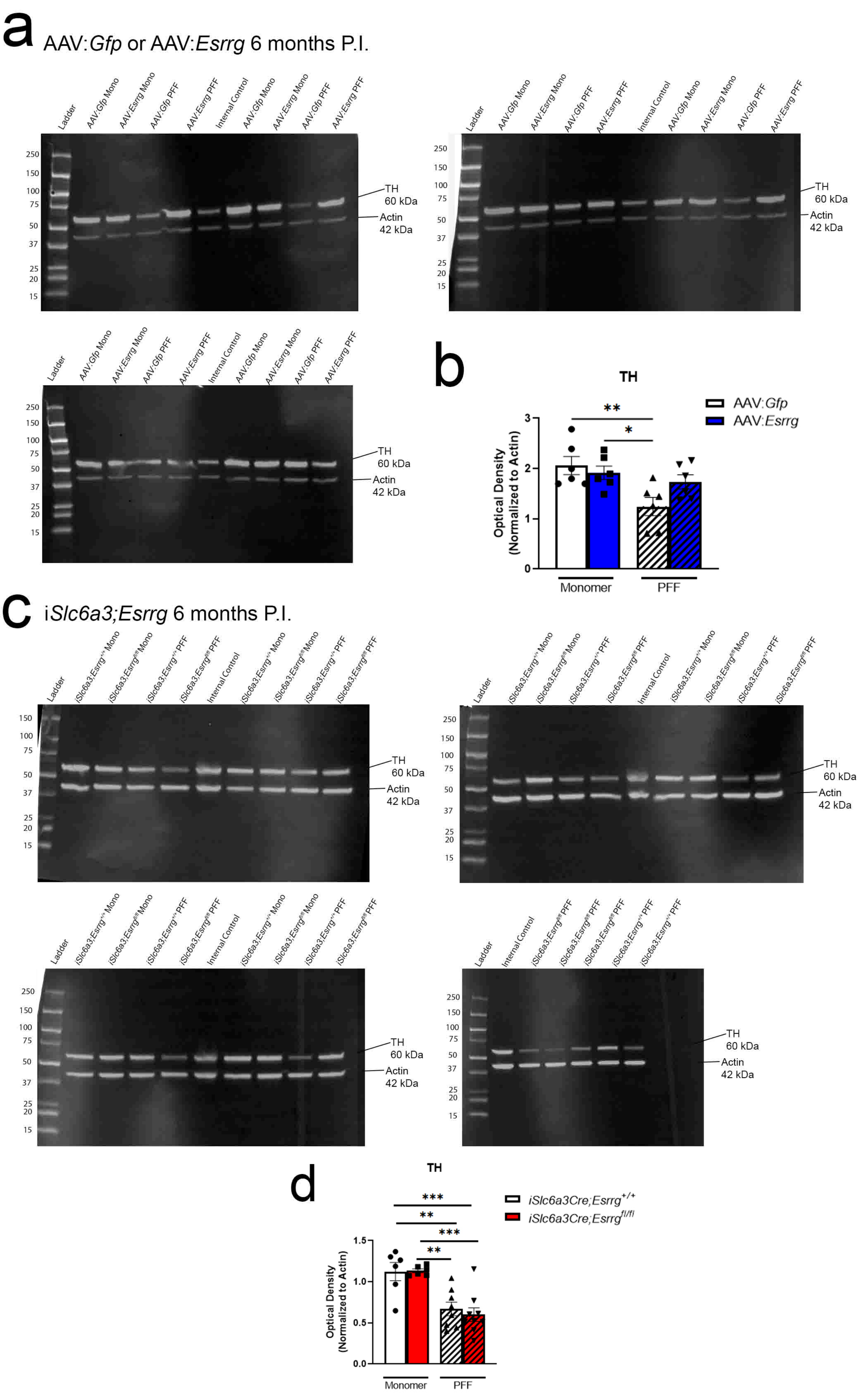

Supplementary Figure 6. a. TH and Actin western blot following AAV-*Gfp* or AAV-*Esrrg* injected with PFF or monomer 6 months post injection with quantification **b.** (*n*=6/group; two-way ANOVA with Tukey's *post-hoc* analysis $*p<0.05$, $**p<0.01$). **c.** TH and Actin western blot from i*Slc6a3Cre;Esrrg*$^{+/+}$ and i*Slc6a3Cre;Esrrg*$^{fl/fl}$ mice 1, 3, and 6 months P.I. with PFFs or monomer with quantification **d.** (*n*=6-9/group; mixed-effects analysis with Sidak's *post-hoc* analysis $**p<0.01$, $***p<0.001$). Error bars represent ±SEM.

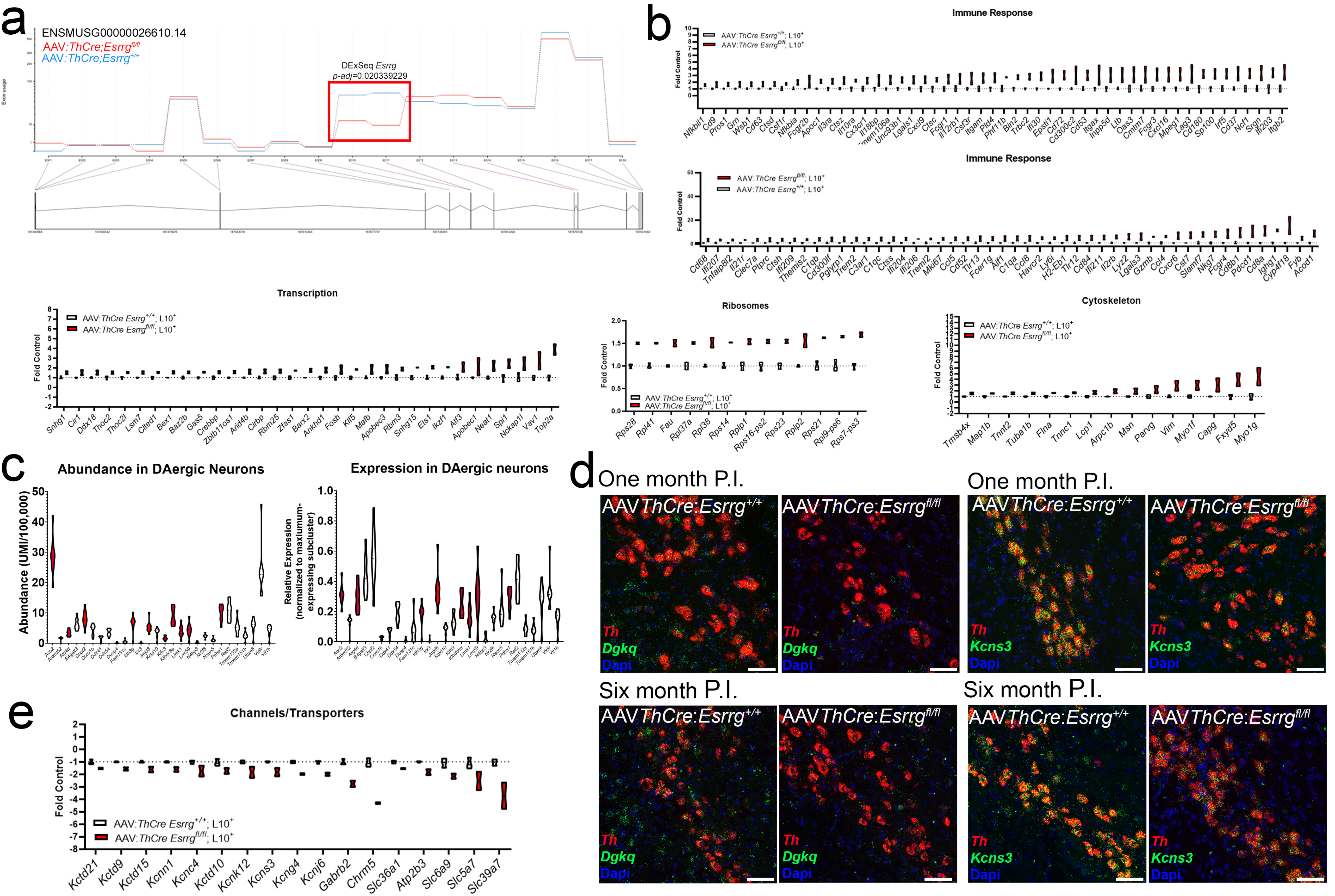

Supplementary Figure 7. a. Splicing model generated from BAC-TRAP DEXseq data; significantly reduced region corresponds to exon 2 of NM_011935.3. **b.** Categories from upregulated genes from BAC-TRAP with *Esrrg* deletion in DAergic neurons. **c.** Abundance of genes found with BAC-TRAP in DAergic neuron populations. Genes measured with rt-qPCR are shown in red. **d.** Representative sm-FISH images for *Kcns3* and *Dgkq* from 1 and 6 months post deletion of *Esrrg* **e.** Fold change of channels and transporters that were reduced with *Esrrg* deletion and found with BAC-TRAP analysis. Scale bars correspond to 50μm (**d**). Error bars represent ±SEM.

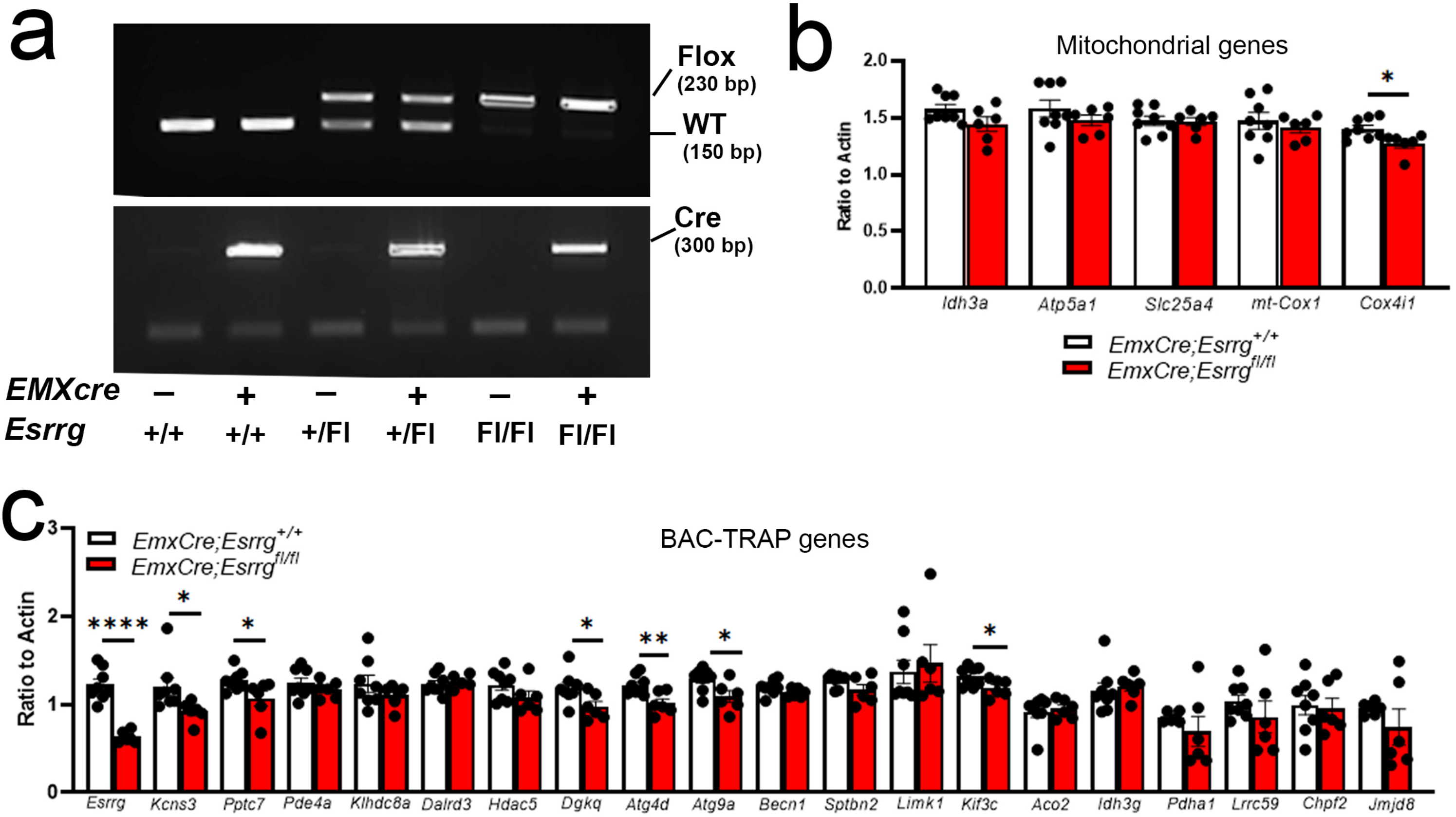

Supplementary Figure 8. a. Sample genotyping gel showing cre- and cre+ *Esrrg*$^{+/+}$, *Esrrg*$^{fl/+}$, and *Esrrg*$^{fl/fl}$ mice. **b, c.** q-rt-PCR from *Emx1Cre* mice crossed with *Esrrg*$^{+/+}$ or *Esrrg*$^{fl/fl}$ mice for BAC-TRAP genes that changed in *Esrrg* midbrain knockout homogenate (**b**) or mitochondrial genes that are known *Esrrg* targets or dependent on *Esrrg* (**c**; *n*=8/group, two-tailed unpaired *t*-tests *p<0.05, **p<0.005). Error bars represent ±SEM.

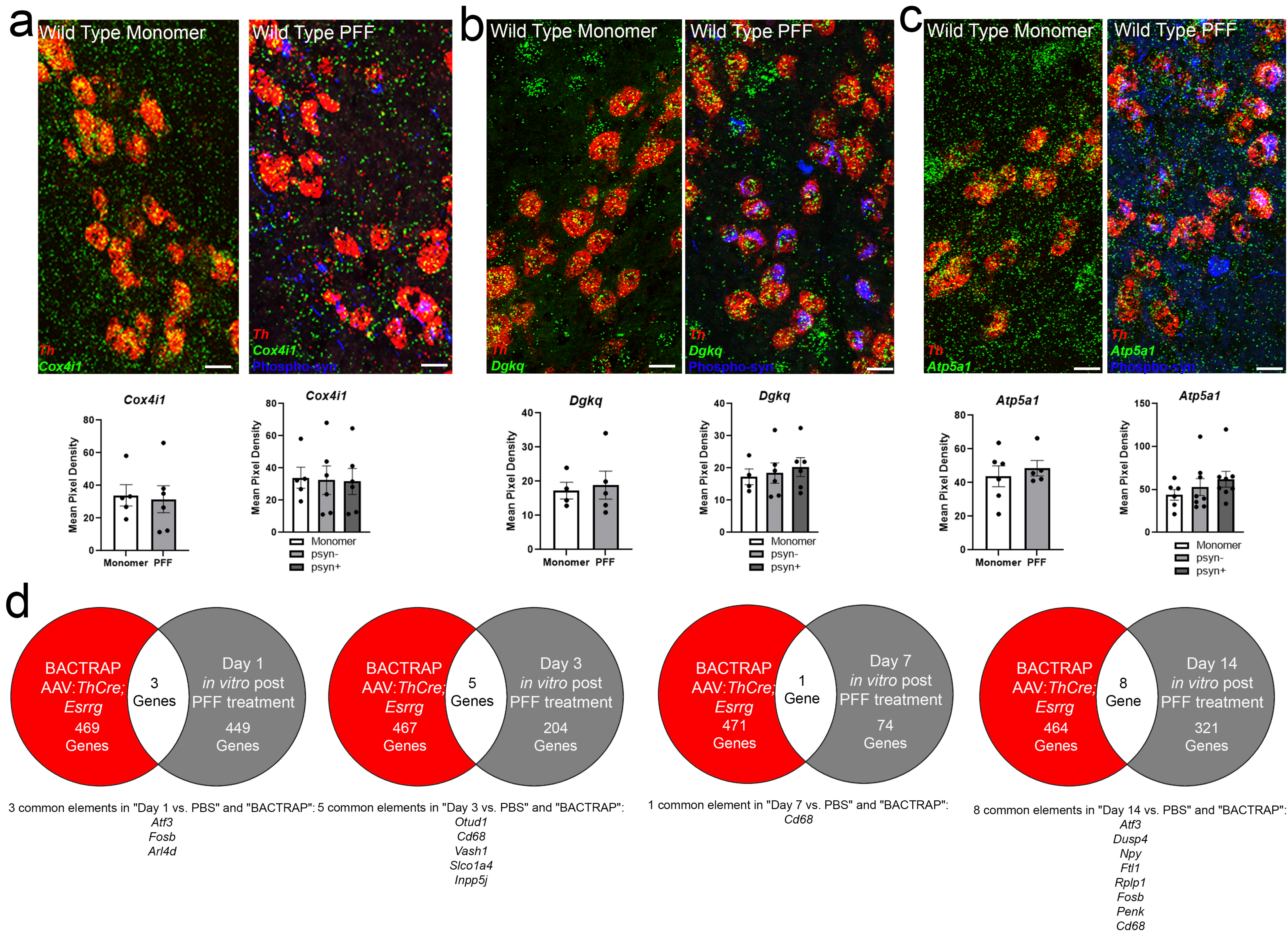

Supplementary Figure 9. a-c. Representative images and sm-FISH analyses of genes in DAergic neurons of wild-type mice injected with PFF or monomer for *Cox4i1*, *Dgkq* and *Atp5a1*. **d.** Pie charts from different time-points (Day 1, 3, 7, 14 and 21) from RNAseq from hippocampal cultured neurons[85] exposed to PFF's and their overlap with genes altered with *Esrrg* deletion specifically from DAergic neurons. Scale bars correspond to 50μm (**a-c**). Error bars represent ±SEM.

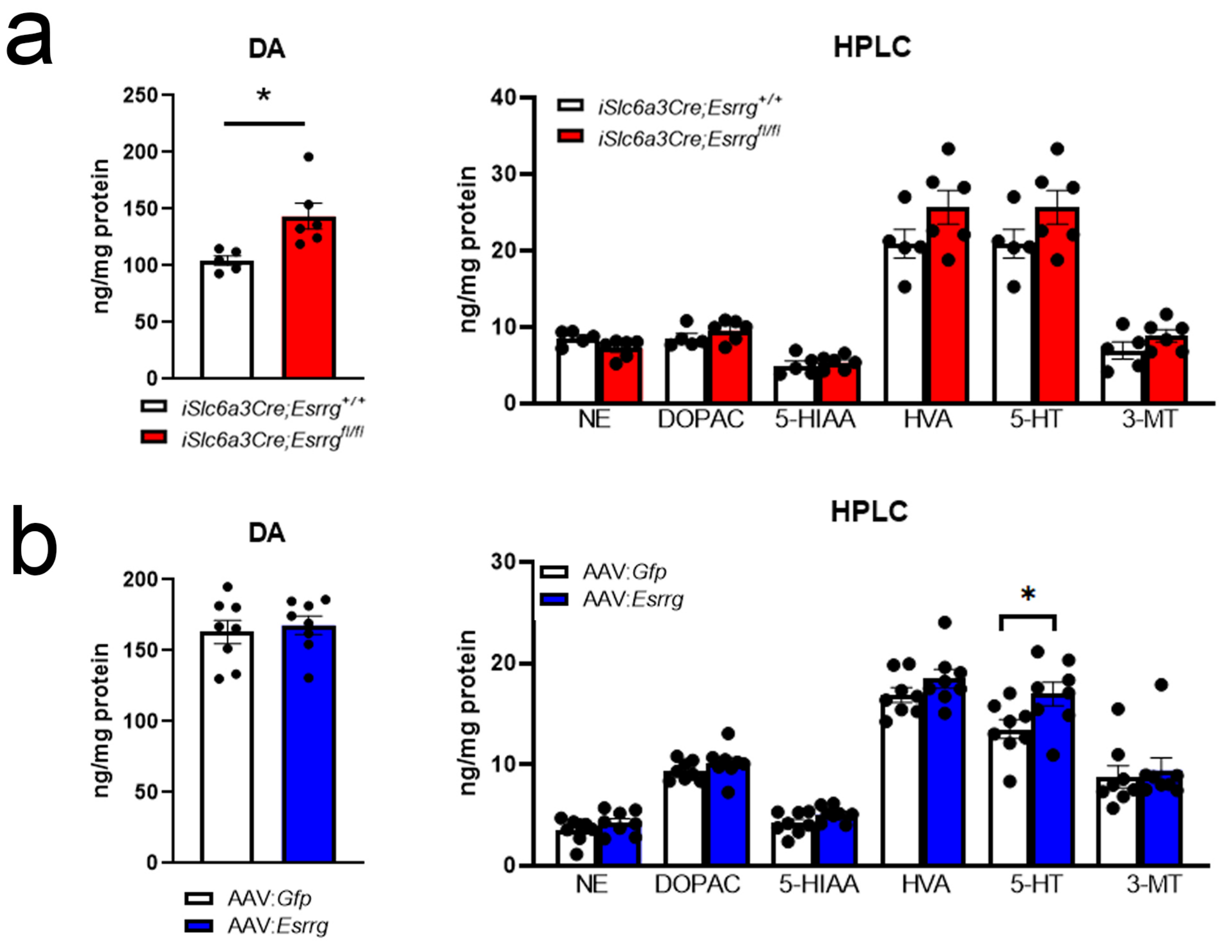

Supplementary Figure 10. a. HPLC data from tamoxifen-injected i*Slc6a3Cre;Esrrg*$^{+/+}$ and i*Slc6a3Cre;Esrrg*$^{fl/fl}$ mice at 9 months post-injection (P.I.) (n=5-6/group; two-tailed unpaired t-tests, *p<0.05). b. HPLC data from the striatum of mice overexpressing *Esrrg* (n=8/group, two-tailed unpaired t-tests *p<0.05). Error bars represent ±SEM.

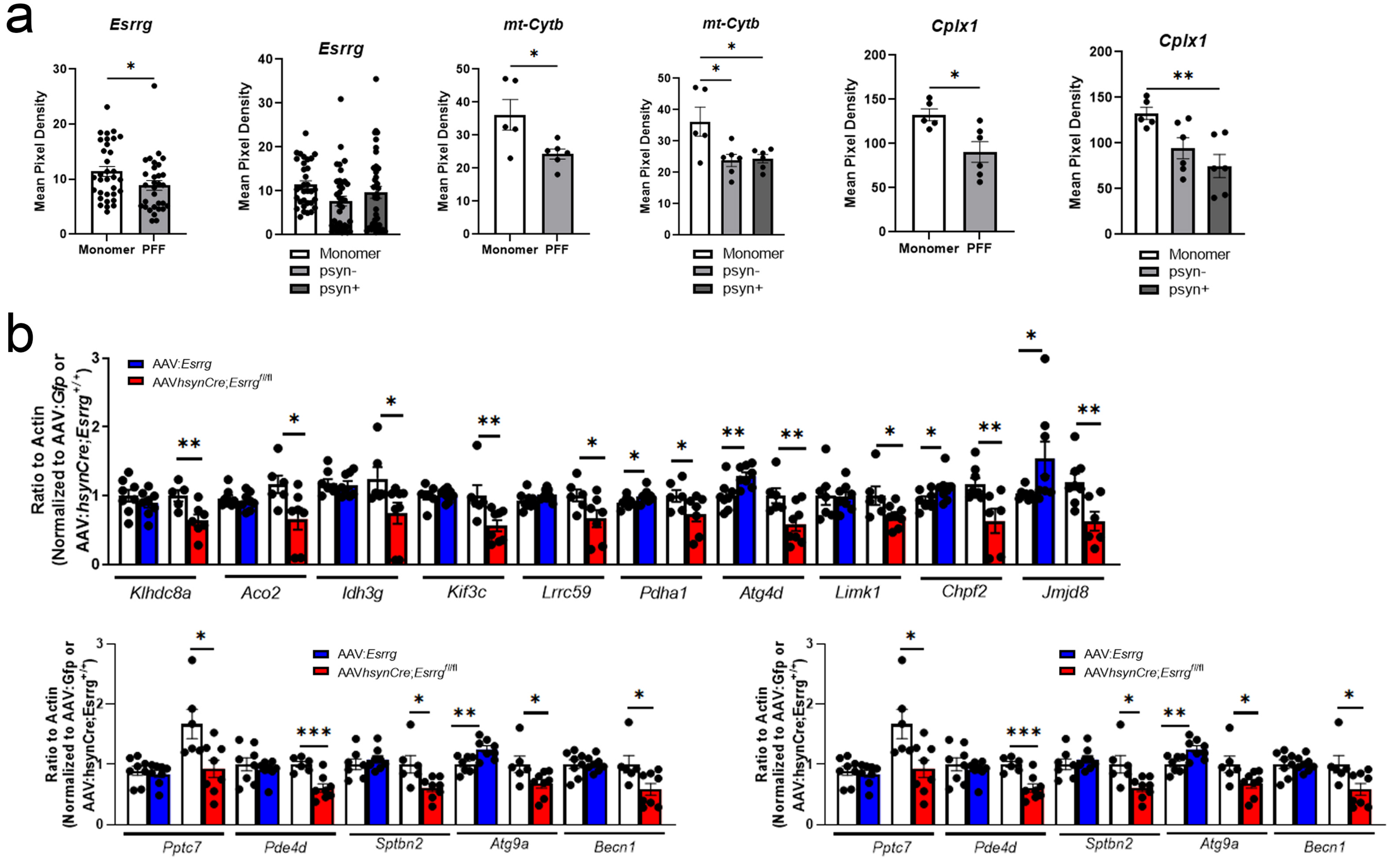

Supplementary Figure 11. Individual datapoints for q-rt-PCR graphs shown in the main manuscript. Error bars represent ±SEM.

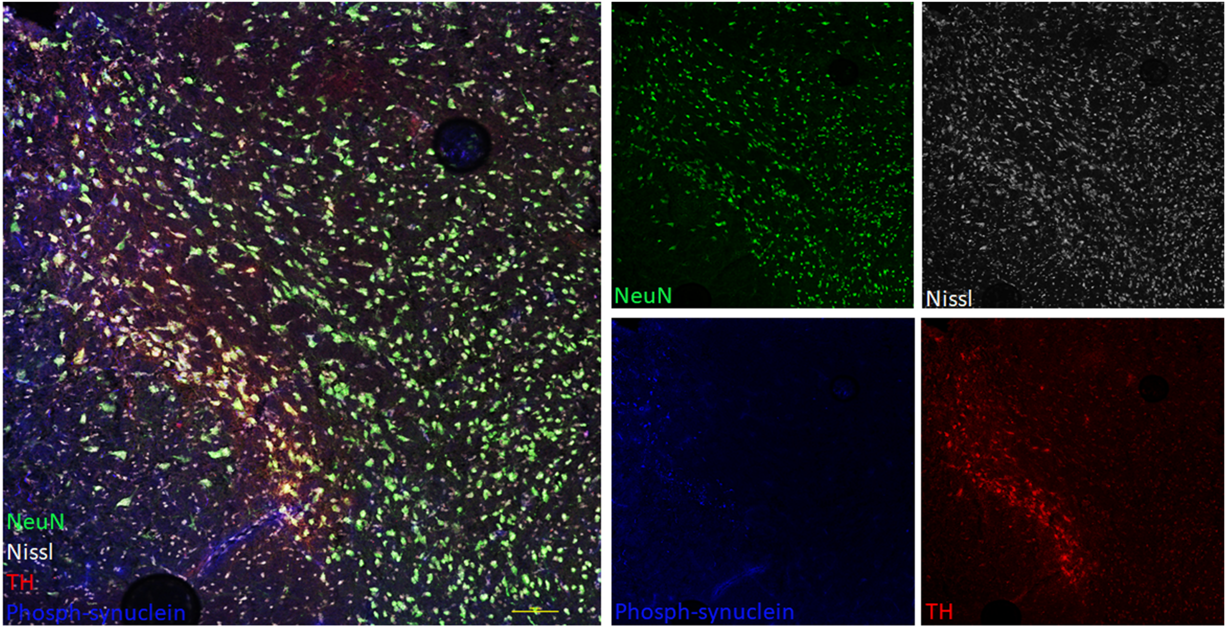

Supplementary Figure 12. Example images from experiments using immunofluorescence for TH (red), NeuN (green), Nissl (white), and phospho-synuclein (blue). Scale bars correspond to 100μm.

Supplementary Table 1. Summary of behavioral results by experiment.

Mouse Model	*Esrrg* (Expression change)	Months Post Deletion	Open Field (Baseline)	Open Field (*d*-AMPH)	Pole Assay	Terminals (TH/DAT)	Cell bodies	Sex differences (in ERRg response)	Sex differences (M vs. F; same group)
AAV:*ThCre* ;*Esrrg* $^{fl/fl}$ vs. AAV:*ThCre;Esrrg* $^{+/+}$	80% reduction	1 month	Hypoactive	Hyperactive	Deficit	No change	No change	No	No
		3 months	Hypoactive	Hypoactive	Deficit	NA	NA	Yes (OF)	Yes (pole)
		6 months	Hypoactive	Hypoactive	Deficit	Decreased	Decreased	Yes (pole)	No
iSlc6a3;Esrrg $^{fl/fl}$ *vs. iSlc6a3;Esrrg+/+*	60% reduction	4 months	Hyperactive	Hyperactive	No change	No change	No change	No	No
		6 months	Hyperactive	No change	No change	No change	No change	No	No
		9 months	Hyperactive	Hyperactive	Deficit	No change	No change	No	No
iSlc6a3;Esrrg fl/fl PFF *vs. iSlc6a3;Esrrg* $^{+/+}$ *PFF*	60% reduction	1 month monomer	Hyperactive	No change	No change	No change	NA	No	No
		1 month PFF	Hyperactive	No change	Deficit	Decreased	NA	No	No
		3 month monomer	No change	No change	No change	No change	No change	No	No
		3 months PFF	No change	No change	Deficit	Decreased	Decreased	No	No
		6 months monomer	No change	Hyperactive	Deficit	No change	No change	No	No
		6 months PFF	No change	No change	Deficit	Decreased	Decreased	No	No
AAV:Esrrg PFF vs. AAV:*Gfp* PFF	10 fold increase	1 month monomer	No change	No change	No change	No change	NA	No	Yes (OF)
		1 month PFF	No change	No change	No change	No change	NA	No	Yes (OF)
		3 month monomer	No change	No change	No change	No change	No change	No	No
		3 months PFF	No change	No change	No change	No change	No change	No	Yes (OF)
		6 months monomer	No change	Hypoactive	No change	No change	No change	No	No
		6 months PFF	No change	No change	No change	No change	No change	No	No

Supplementary Table 2. Annotation of ERRA chromatin immunoprecipitation sites in ERRg-dependent genes from the UCSC Genome Browser.

28 overlap between FLAG seq and BACTRAP	Promoter ERRa ChIP (GRCh38/hg38)	Intragenic ERRa ChIP	Genes with no evidence of ERRa ChIP binding
ACO2	chr22:41468857-41469766	chr22:41523605-41524068	AIF1
ANKRD52	chr12:56261870-56262205; chr12:56258110-56258516		ATP2B3
ATG4D	chr19:10541452-10541915; chr19:10543872-10545002		BRSK1
B4GALT3	chr1:161177531-161178022		CHRNA7
CHPF2	chr7:151232339-151232543		CLVS1
CORO1B	chr11:67449378-67449841		DHX32
DDX41	chr5:177516884-177517358		DOC2A
DDX54	chr12:113185952-113186465	chr12:113181946-113182409	FAM13C
DUSP4		chr8:29340187-29340650	FGF11
FAM131C	chr1:16077000-16077335; chr1:16073561-16074024	chr1:16070607-16071020; chr1:16069044-16069159; chr1:16067432-16067590; chr1:16057612-16057905	GABRB2
IDH3G	chrX:153794250-153794713; chrX:153793176-153793850		Ifi203
IRX3	none		IRX3
JMJD8	chr16:684275-684738		KCNS3

KCTD10	chr12:109477298-109477761	chr12:109461111-109461574	LGALS3
KIFC3	chr16:57828810-57829027; chr16:57808416-57808879; chr16:57810412-57810648; chr16:57805194-57805437; chr16:57801803-57802046; chr16:57801344-57801587; chr16:57800500-57801157	chr16:57765253-57765716	NPY
KLHDC8A	chr1:205357501-205357964	chr1:205344281-205344616; chr1:205335560-205336023	PENK
LIMK1	chr7:74084297-74084760		PTGR2
LRRC59	chr17:50397455-50397697		RIMKLA
N4BP3	chr5:178106972-178107307; chr5:178108011-178108346; chr5:178113344-178113749; chr5:178113946-178114171	chr5:178116283-178116683; chr5:178117194-178117529; chr5:178120880-178121545; chr5:178123874-178124193; chr5:178124265-178124881; chr5:178127531-178127994	RTKN

NR2F6	chr19:17248002-17248550; chr19:17246367-17247030; chr19:17244603-17245205	chr19:17235408-17235871	Slco1a4
NSUN5	chr7:73308692-73309072		SMYD2
PDHA1	chrX:19343759-19344243		TGFBI
RELL2	chr5:141636701-141636836		TMEM132A
TMEM132A	none		TSPO
TMEM151B	chr6:44268000-44268463		
UBXN6	chr19:4457789-4458252		
VLDLR	chr9:2602549-2602884		
YIF1B	chr19:38317041-38317707; chr19:38315632-38316024	chr19:38304077-38304412; chr19:38300774-38301109; chr19:38299328-38299663	

**44 common elements in "PGC1a up" and "Bactrap Down":**	**Promoter ERRa ChIP (GRCh38/hg38)**	**Intragenic ERRa ChIP**
ACO2	chr22:41468857-41469766	chr22:41523605-41524068
ADCK2	chr7:140675226-140675689	
ATG4D	chr19:10541452-10541915; chr19:10543872-10545002	
ATG9A	chr2:219228992-219229347	
ATP2B3	none	

ATPAF1	chr1:46668330-46668447	chr1:46637605-46638068
BRSK1	none	
CABLES2	chr20:62409954-62410289; chr20:62407293-62407955; chr20:62406550-62407013	
CLK3	chr15:74615545-74616207	
CYB5D1	chr17:7857070-7857425	
DHX32	none	
DOC2A	none	
EPHA8	chr1:22537705-22538168; chr1:22560388-22560851	chr1:22573196-22573659
FAM131C	chr1:16077000-16077335; chr1:16073561-16074024	chr1:16070607-16071020; chr1:16069044-16069159; chr1:16067432-16067590; chr1:16057612-16057905
FER1L5		chr2:96688898-96689176
FGF11	none	
GABRB2	none	
HMOX2	chr16:4474394-4474857	
IDH3G	chrX:153794250-153794713; chrX:153793176-153793850	
INPP5J	chr22:31119144-31119607; chr22:31122060-31122523	chr22:31122060-31122523; chr22:31134701-31134979
IQSEC1	24 sites (see UCSC)	

KCNN1	chr19:17950157-17950848; chr19:17953058-17953726; chr19:17953887-17954350	chr19:17969933-17970253; chr19:17977498-17977961; chr19:18001040-18001503
KCNS3	none	
KCTD9	chr8:25458880-25459343	
KLHDC8A	chr1:205357501-205357964	chr1:205344281-205344616; chr1:205335560-205336023
N4BP3	chr5:178106972-178107307; chr5:178108011-178108346; chr5:178113344-178113749; chr5:178113946-178114171	chr5:178116283-178116683; chr5:178117194-178117529; chr5:178120880-178121545; chr5:178123874-178124193; chr5:178124265-178124881; chr5:178127531-178127994
NCOA4	chr10:46031772-46032527; chr10:46030441-46030992	chr10:46023173-46023755; chr10:46020289-46020735
PDHA1	chrX:19343759-19344243	
PDPR	chr16:70098897-70099360; chr16:70114026-70114489	chr16:70136730-70136856

PDXK	chr21:43698507-43698970; chr21:43702905-43703368; chr21:43712221-43712556; chr21:43719058-43719774	
PLEKHH1	chr14:67524667-67524831	
PPTC7	chr12:110602045-110602508; chr12:110591573-110591908; chr12:110583110-110583465; chr12:110582338-110582900	chr12:110577712-110578339; chr12:110563917-110564380
PTGR2	none	
RIMKLA	none	
SACM1L	chr3:45688934-45689363	
SIRT3	chr11:236669-237132	chr11:220621-220920; chr11:217610-218073
SLC39A7	chr6:33200174-33200391; chr6:33200523-33201561	
SMYD2	none	
THTPA	chr14:23555911-23556466	
TM6SF1	chr15:83086100-83086563	chr15:83111217-83111536; chr15:83113026-83113361
TMEM132A	none	
TMEM151B	chr6:44268000-44268463	
TMEM184C	chr4:147617228-147617691	

YIF1B	chr19:38317041-38317707; chr19:38315632-38316024	chr19:38304077-38304412; chr19:38300774-38301109; chr19:38299328-38299663

23 common elements in "Day 21 PFF" and "Bactrap":

Atf3	chr1:212558099-212559111	chr1:212587581-212588044; chr1:212605710-212606173; chr1:212608675-212608821
Cd68	chr17:7579394-7579895	chr17:7581014-7581477
Chrna7	none	
Clvs1	none	
DUSP4		chr8:29340187-29340650
Fam13c	none	
Flna	chrX:154379160-154379539; chrX:154370910-154371373	chrX:154358870-154359205; chrX:154358207-154358493; chrX:154357088-154357423; chrX:154347245-154347580
Fosb	chr19:45464175-45464638; chr19:45467284-45467747	
Ftl1	chr19:48965161-48965624	
Ifi203	no human homolog	

Igfbp2	chr2:216626249-216626584	
Kcnj6	chr21:38166963-38167426; chr21:38171773-38172108; chr21:38163530-38163849; chr21:38121303-38121766; chr21:38134783-38135102; chr21:38112911-38113374	chr21:37768177-37768263; chr21:37656449-37656912
KIFC3	chr16:57828810-57829027; chr16:57808416-57808879; chr16:57810412-57810648; chr16:57805194-57805437; chr16:57801803-57802046; chr16:57801344-57801587; chr16:57800500-57801157	chr16:57765253-57765716
KLHDC8A	chr1:205357501-205357964	chr1:205344281-205344616; chr1:205335560-205336023
Npy	none	
Penk	none	
Rtkn	none	
S100a11	chr1:152036841-152037304; chr1:152036075-152036394	chr1:152035059-152035394
Slco1a4	no human homolog	

Sptbn2		chr11:66709829-66710164; chr11:66701835-66702079; chr11:66694694-66695157
Tgfbi	none	
Tspo	none	
Vim	chr10:17228482-17228945; chr10:17230331-17230794	

15 common elements in "PD GWAS + QTL" and "Bactrap":

Aif1	none	
Arel1	chr14:74712918-74713381; chr14:74710451-74710914	chr14:74649492-74649827
Dalrd3	chr3:49021740-49022232	
Dgkq	chr4:973721-974184	chr4:971766-972101
FGF11	none	
Grn	chr17:44348392-44348747	chr17:44350693-44351156
Hdac5	chr17:44132503-44132838; chr17:44123608-44124453	chr17:44111749-44112212; chr17:44111058-44111521; chr17:44103085-44103420; chr17:44095747-44096210
Kcns3	none	
Lgals3	none	
Lsm7	chr19:2328518-2328981	
Mcm6	chr2:135853409-135853799	

Nupr1	chr16:28538811-28539274	
Plekhh1	chr14:67524667-67524831	
Rab7b	chr1:206000601-206000936	chr1:205983895-205984509; chr1:205977401-205977864
Rnf123	chr3:49689321-49689784	

Supplementary Table 3. sm-FISH probes from ACDBio.

Gene name	Species	Catalog number
ESRRG	Human	523271
TH	Human	441651
Esrrg Basescope	Mouse	861621
Esrrg	Mouse	495121
Th	Mouse	317621
Aldh1a1	Mouse	491321
mt-Cytb	Mouse	517301
Atp5a1	Mouse	459311
Cox4i1	Mouse	538161
Dgkq	Mouse	534851
Kcns3	Mouse	467371
Cplx1	Mouse	482531

Supplementary Table 4. Antibody information.

Protein name	Species	Antibody manufacturer and catalog number
TH	Rabbit	Millipore Sigma Cat # AB152
DAT	Rat	Millipore Sigma Cat # MAB269
phospho-synuclein	Mouse	Biolegend Cat # 825701
NeuN	Chicken	EMD Millipore Cat# ABN91
Nissl	N/A	Thermo Fisher Scientific Cat#N21483
Actin	Mouse	Millipore Signma Cat# MAB1501

Supplementary Table 5. Post Mortem Tissue Information.

Alabama Brain Collection case	Age Race Sex	Postmortem Interval hours	pH	Cause of death	Smoker	Diabetes
1	67 white male	15	6.4	Lung cancer	smoker	No
2	53 white male	33	6.3	Acute myocardial infarction	smoker	No
3	70 white male	9	6.4	myocardial infarction	smoker	No

Supplementary Table 6. Inventoried Primer/Probe sets from Taqman (Applied Biosystems).

Gene name	Taqman gene expression assay Code
Actin	Mm00607939_s1
Esrrg	Mm01318906_m1
Esrra	Mm00433143_m1
Ppargc1a	Mm01208835_m1
Tfam	Mm00447485_m1
Atp5a1	Mm00431960_m1
Cox4i1	Mm01250094_m1
Slc25a4	Mm01207393_m1
Idh3a	Mm00499674_m1
mt-Cox1	Mm04225243_g1
Slc18a2	Mm00553058_m1
Cplx1	Mm00514378_m1
Snca	Mm01188700_m1
Sncb	Mm00504325_m1
Sncg	Mm00488345_m1
Comt	Mm00514377_m1
Maoa	Mm00558004_m1
Maob	Mm00555412_m1
Th	Mm00447557_m1
Ddc	Mm00516688_m1
Gad65	Mm00484623_m1
Klhdc8a	Mm00522717_m1
Aco2	Mm00475673_g1
Idh3g	Mm00599689_m1
Kif3c	Mm00492900_m1
Lrrc59	Mm00505480_m1
Pdha1	Mm00468675_m1
Atg4d	Mm00463819_m1
Limk1	Mm00440194_m1
Chpf2	Mm00659795_m1
Jmjd8	Mm00511259_g1
Dalrd3	Mm00509493_g1
Arel1	Mm00626177_m1
Kcns3	Mm01272234_s1
Hdac5	Mm00515917_m1
Dgkq	Mm01198794_m1
Pptc7	Mm00623697_m1
Pde4d	Mm004556879_m1
Sptbn2	Mm01239117_m1
Atg9a	Mm01264420_m1
Becn1	Mm012565461_m1
